# Transplantation and Surgical Strategies in Patients With Neuroendocrine Liver Metastases: Protocol of Four Systematic Reviews

**DOI:** 10.2196/resprot.2891

**Published:** 2013-12-23

**Authors:** Reto Stump, Silvia Haueis, Nicola Kalt, Christoph Tschuor, Përparim Limani, Dimitri A Raptis, Milo A Puhan, Stefan Breitenstein

**Affiliations:** ^1^Division of Visceral and Transplantation SurgeryDepartment of SurgeryUniversity Hospital ZurichZurichSwitzerland; ^2^Institute for Social and Preventive MedicineUniversity of ZurichZurichSwitzerland; ^3^Department of SurgeryCantonal Hospital WinterthurClinic for Visceral and Thoracic SurgeryWinterthurSwitzerland

**Keywords:** neuroendocrine tumors, NET, liver resection, adjuvant neoadjuvant, liver transplantation, primary NET, systematic review

## Abstract

**Background:**

Hepatic metastases of neuroendocrine tumors (NETs) are considered a major prognostic factor associated with significantly reduced survival compared to patients without liver metastases. Several surgical and nonsurgical strategies are present to treat resectable and nonresectable liver metastases, some of which have the potential to cure liver mestatases.

**Objective:**

The aims of the four systematic reviews presented in the paper are to determine the effectiveness of liver resection versus nonsurgical treatment of patients with NET liver metastases, to investigate the impact of neoadjuvant and adjuvant treatment options on the tumor-free survival, to assess the role of liver transplantation in patients presenting with unresectable bilateral hepatic metastases, and to evaluate the role of primary tumor resection in presence of unresectable liver metastases.

**Methods:**

Literature search was performed on Medical Literature Analysis and Retrieval System Online, Excerpta Medica Database, and the Cochrane Library (Cochrane Database of Systematic Reviews, Database of Abstracts of Reviews of Effects, and Cochrane Central Register of Controlled Trials). No language restrictions were applied. Randomized controlled trials, prospective and retrospective comparative cohort studies, and case-control studies will be used for the qualitative and quantitative synthesis of the systematic reviews. Case series will be only included in a separate database for descriptive purposes.

**Results:**

This study is ongoing and presents a protocol system of four systematic reviews that will assist in determining the effectiveness of liver resection versus nonsurgical treatment of patients with NET liver metastases. This study is also assumed to investigate the impact of neoadjuvant and adjuvant treatment options on the tumor-free survival, the role of liver transplantation, and the relevance of primary tumor resection in presence of unresectable liver metastasis.

**Conclusions:**

The systematic reviews will show the current evidence based on the effectiveness of surgical strategies in patients with NET liver metastases and serve as basis for clinical practice guidelines.

**Trial Registration:**

The systematic reviews have been prospectively registered with the International Prospective Register of Systematic Reviews: liver resection (CRD42012002652); http://www.crd.york.ac.uk/prospero/display_record.asp?ID=CRD42012002652 (Archived by WebCite at http://www.webcitation.org/6LQUqMnqL,). neoadjuvant and adjuvant treatment strategies (CRD42012002656); http://www.crd.york.ac.uk/prospero/display_record.asp?ID=CRD42012002656 (Archived by WebCite at http://www.webcitation.org/6LQVvEHuf). liver transplantation (CRD42012002655); http://www.crd.york.ac.uk/prospero/display_record.asp?ID=CRD42012002655 (Archived by WebCite at http://www.webcitation.org/6LQW7WFo3,). resection of the locoregional primary NET (CRD42012002654); http://www.crd.york.ac.uk/prospero/display_record.asp?ID=CRD42012002654 (Archived by WebCite at http://www.webcitation.org/6LQWEIuGe).

## Introduction

### Background

#### Neuroendocrine Tumors

Neuroendocrine tumors (NETs) developing from neuroendocrine cells can originate almost everywhere in the body [1]. Primary NETs are mainly located in the bronchopulmonary (>25%) and the gastroenteropancreatic system (60%) [2,3]. With an annual age-adjusted incidence of 5.25 cases per 100,000 people, NETs are considered to be rare tumors. Most NETs occur sporadically, whereas a minority of cases may develop due to genetic syndromes such as multiple endocrine neoplasia type 1 [4].

According to their functional behavior, NETs can be subdivided into two categories: functioning NETs and nonfunctioning NETs [5]. Functioning NETs secrete specific products such as biogenic amines and polypeptide hormones and can cause endocrine syndromes such as the carcinoid syndrome. Endocrine syndromes in tumors with portal venous drainage often begin in the presence of liver metastases. Metastases drain active hormones directly into the systemic circulation while the liver metabolizes hormones derived from primary tumors [6,7]. Therefore, functioning tumors are usually detected earlier than nonfunctioning tumors and patients seem to have a better overall survival (OS) [8]. The nonfunctioning NETs may cause local tumor mass-related symptoms or are found incidentally [7,9].

#### Liver Metastases of NETs

Despite the slow growing nature of NETs, Pape et al reported liver metastases of gastroenteropancreatic NETs in 84.7% of cases at the initial diagnosis [10]. Due to the favorable environment, metastases of NETs are confined to the liver for a prolonged period of time [11]. Hepatic metastases are considered to be a major prognostic factor, associated with a significantly reduced survival compared to patients without liver metastases [12,13]. Furthermore, the metastatic pattern within the liver also has prognostic and therapeutic impact. Frilling et al suggested three different patterns of liver metastases: single metastasis of any size (type 1); isolated metastatic bulk accompanied by smaller deposits, with both liver lobes always involved (type 2); and disseminated metastatic spread, with both liver lobes always involved, single lesion of varying size and virtually no normal liver parenchyma (type 3). This classification is believed to represent differences in biologic characteristics of the tumors, which require different treatment strategies [14].

#### Liver Resection

A wide array of options is available to treat liver metastases from NETs, which improves the 5-year OS in 60-80% patients who have undergone curative surgery compared to less than 40% untreated patients [15-17]. Surgical interventions contain potentially curative resection of the metastases (R0/R1). If R0/R1 resection is not feasible, a palliative resection is performed in patients suffering from tumor bulk or hormonal symptoms, especially in patients with functioning NETs and who are unresponsive to treatment. However, guidelines suggest that palliative surgery should only be performed if at least 90% of the metastatic bulk can be safely removed [18]. Curative resection can only be achieved in patients with a metastatic pattern type 1, while patients with type 2 or 3 need to be evaluated for other treatment options [14]. Therefore, curative resection is only feasible in less than 20% of patients due to the high rate of diffuse and bilobar spreading of metastases [19]. For patients with a metastatic pattern type 2 or 3, there are several locoregional techniques such as radiofrequency ablation, transcatheter arterial chemoembolization (TACE), and systemically applied therapies (eg, chemotherapy or peptide receptor radionuclide therapy [PRRT]) [17]. The first systematic review intends to compare curative and palliative liver resection versus or in combination with nonsurgical treatment options.

#### Neoadjuvant and Adjuvant Treatment Options

Disease recurrence after surgical treatment of liver metastases is often observed, even when resection is performed with curative intent [20]. To increase the resectability and to reduce the high rate of metastatic relapse, neoadjuvant and adjuvant treatment options need to be evaluated. According to their treatment modality, neoadjuvant and adjuvant treatment options can be divided into systemic (chemotherapy, biotherapy, and PRRT) and liver-directed therapies (selective internal radiation therapy [SIRT], transcatheter arterial embolization [TAE], and TACE). For the chemotherapeutical strategy, several substances have been used to treat NETs, either as a monotherapy or combined in different regimens [21-23]. Biotherapy for NETs essentially includes treatment with somatostatin analogues, such as octreotide and lanreotide, in order to control hormone-related symptoms [24]. PRRT consists of systemically applied radiolabeled somatostatin derivates that bind specifically to the somatostatin receptor, which is overexpressed in certain NETs and thereby damage the tumor cell [25]. Liver-directed techniques, such as SIRT, TAE, and TACE, make use of the biologic feature that hepatic neoplasms are preferentially supplied via the hepatic artery, whereas normal liver parenchyma is mainly supplied by the portal vein [26,27].

For liver metastases arising from a non-NET primary tumor, the benefit of neoadjuvant and adjuvant strategies combined with liver resection has already been investigated in more detail [28,29]. Nordlinger et al reported that the risk of recurrent disease in patients with liver metastases of colorectal carcinomas could be reduced compared to surgical resections alone [29]. Adopting these strategies to the treatment of NET liver metastasis could be a promising option. The second systematic review intends to evaluate whether neoadjuvant and/or adjuvant treatment strategies together with surgical resection are superior to liver resection alone.

#### Liver Transplantation

Controversy concerning liver metastases from NETs as an indication for liver transplantation arises inter alia from the relatively low number of such patients being transplanted. Moreover, heterogeneous 5-year OS data have been published with ranges between 33% and 96% [16,30]. Therefore, our third systematic review aims to evaluate the possible benefit of liver transplantation as a treatment option for unresectable hepatic metastases of NETs and to define selection criteria to choose patients with the best possible prognosis.

#### Resection of the Primary NET

Another important question is whether the primary tumor should be removed in presence of nonresectable liver metastases as the answer may improve the outcome. Potential benefits of resection are seen in providing relief from hormonal and local tumor mass-related symptoms [31]. Since evidence is missing, the fourth systematic review aims to answer this question.

### Objective

The purpose of these four systematic reviews is to assess the role of surgical strategies in the management of liver metastases of nets, to evaluate the use of adjuvant and neoadjuvant therapies, to define selection criteria for patients who benefit the most from liver transplantation, and to study the influence of resection of the primary tumor.

## Methods

### Overview

These four systematic reviews dealing with surgical treatment options for NET liver metastases attempt to answer the questions with regard to liver resection in patients with hepatic metastases (see Textbox 1), neoadjuvant and adjuvant treatment strategies (see Textbox 2), liver transplantation in patients with unresectable hepatic metastases (see Textbox 3), and resection of the locoregional primary neuroendocrine tumor (see Textbox 4).

We will report our review findings in accordance with the standards of the Preferred Reporting Items for Systematic reviews and Meta-Analyses [32]. Our reviews were prospectively registered with the International Prospective Register of Systematic Reviews: liver resection (CRD42012002652) [33], neoadjuvant and adjuvant treatment strategies (CRD42012002656) [34], liver transplantation (CRD42012002655) [35], and resection of the locoregional primary NET (CRD42012002654) [36].

**Figure 1:**
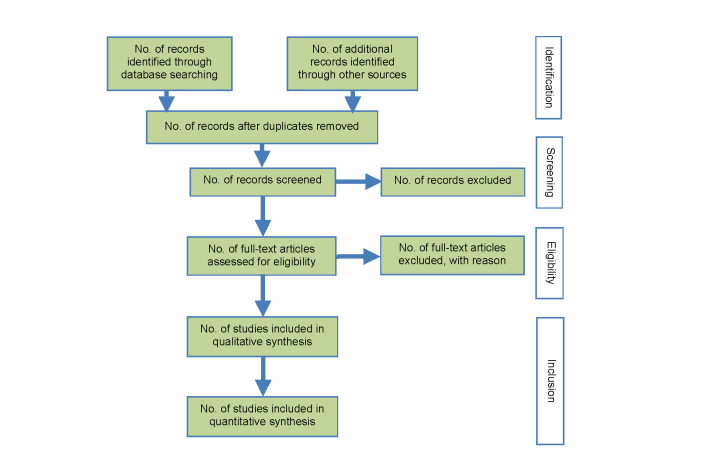
Flow diagram representing the number of excluded studies and reasons for exclusion.

The systematic review inclusion and exclusion criteria are listed in Tables 1-4. No language or publication date restrictions were imposed on the literature search. All accessible publications were included. The following study designs will be included for the qualitative synthesis of the systematic review: randomized controlled trials (RCTs), prospective and retrospective comparative cohort studies, and case-control studies. Case series will only be included in a separate database for descriptive purposes. The number of excluded studies and reasons for exclusion will be reported in a flow diagram, according to the PRISMA Statement 2009 (Figure 1) [32].

**Questions with regard to liver resection in patients with hepatic metastases from neuroendocrine tumors.**

In patients with resectable NET liver metastases, does liver resection with a curative intent (R0/R1) improve outcome (tumor-free survival, overall survival, quality of life) when compared to non-surgical treatment (locally ablative techniques, percutaneous liver-directed techniques, peptide receptor radionuclide treatment, chemotherapy, targeted therapy, biotherapy)?

In patients with NET liver metastases, does R2 liver resection (debulking) improve outcome (progression-free survival, overall survival, quality of life) when compared to non-surgical treatment (locally ablative techniques, percutaneous liver-directed techniques, peptide receptor radionuclide treatment, chemotherapy, targeted therapy, biotherapy)?

In patients with NET liver metastases, do locally ablative techniques as an adjunct to R2 liver resection improve outcome (progression-free survival, overall survival, quality of life)?

**Questions with regard to neoadjuvant and adjuvant treatment strategies be used together with liver resection for neuroendocrine liver metastases.**

In patients with NET liver metastases, does neoadjuvant treatment improve outcome (increase in R0/R1 resectability, tumor-free survival, overall survival, quality of life) after liver resection compared to no neoadjuvant treatment?

In patients with NET liver metastases, does adjuvant treatment improve the outcome (tumor-free survival, overall survival, quality of life) of liver resection as opposed to no adjuvant treatment?

In patients with NET liver metastases, do both neoadjuvant and adjuvant treatment strategies improve the outcome (tumor-free survival, overall survival, quality of life) of liver resection compared to no neoadjuvant and adjuvant treatment?

**Questions with regard to liver transplantation in patients with unresectable hepatic metastases from neuroendocrine tumors.**

In patients with non-resectable NET liver metastases, does liver transplantation improve outcome (disease-free / progression-free survival, overall survival, quality of life) as opposed to R2 liver resection (debulking) or non-surgical treatment (locally ablative techniques, percutaneous liver-directed techniques, peptide receptor radionuclide treatment, chemotherapy, targeted therapy, biotherapy)?

In patients with NET liver metastases, which selection criteria should be used for liver transplantation in order to improve outcome (disease-free survival, overall survival, quality of life)?

In patients with NET liver metastases and consideration for liver transplantation, does a delay (≥6 months) to assess tumor progression before transplanting improve the selection of patients (disease-free survival, overall survival, quality of life) as opposed to early transplantation (<6 months)?

In patients with NET liver metastases listed for liver transplantation, does downstaging (locally ablative techniques, percutaneous liver-directed techniques, peptide receptor radionuclide treatment, chemotherapy, targeted therapy, biotherapy) improve outcome (tumor-free survival, overall survival, quality of life)?

In patients with non-resectable NET liver metastases, does living donor liver transplantation improve outcome (disease-free survival, overall survival, quality of life) as opposed to deceased-donor transplantation or non-surgical treatment (locally ablative techniques, percutaneous liver-directed techniques, peptide receptor radionuclide treatment, chemotherapy, targeted therapy, biotherapy)?

Does the outcome of the recipient justify the risk of the donor in the setting of liver transplantation for NET liver metastases?

**Questions with regard to resection of the locoregional primary neuroendocrine tumor in the presence of nonresectable liver metastases.**

In patients with a pancreatic primary NET and non-resectable liver metastases, does resecting the primary tumor improve outcome (progression-free survival, overall survival, quality of life) when compared to non-surgical treatment (peptide receptor radionuclide treatment, chemotherapy, biotherapy)?

In patients with an intestinal primary NET and non-resectable liver metastases, does resecting the loco-regional primary tumor improve outcome (progression-free survival, overall survival, quality of life) when compared to non-surgical treatment (peptide receptor radionuclide treatment, chemotherapy, biotherapy)?

In patients with a lung primary NET and non-resectable liver metastases, does resecting the primary tumor improve outcome (progression-free survival, overall survival, quality of life) when compared to non-surgical treatment (peptide receptor radionuclide treatment, chemotherapy, biotherapy)?

**Table 1:** Eligibility criteria for review on liver resection [33].

Study characteristics	Inclusion criteria	Exclusion criteria
Patient population	Patients with neuroendocrine tumor (NET) liver metastases	Children or adolescents (under the age of 18 years)
	Patients who underwent liver resection or nonsurgical treatment (peptide receptor radionuclide treatment (PRRT), chemotherapy, biotherapy)	
Intervention: treatment	Liver resection	
	Nonsurgical treatment (chemotherapy, biotherapy, locally ablative techniques, radionuclide therapy)	
Intervention: comparison	Liver resection vs nonsurgical treatment (chemotherapy, biotherapy, locally ablative techniques, radionuclide therapy)	
Outcomes	Primary outcome: overall survival (OS)	Studies that do not report the OS
	Secondary outcomes: progression-free survival, quality of life	
Study design	Randomized controlled trials	Case reports
	Prospective and retrospective comparative cohort studies	
	Case-control studies	
	Case series	

**Table 2:** Eligibility criteria for review on neoadjuvant and adjuvant treatments [34].

Study characteristics	Inclusion criteria	Exclusion criteria
Patient population	Patients with neuroendocrine tumor (NET) liver metastases who underwent liver resection with or without neoadjuvant or adjuvant treatment	Children or adolescents (under the age of 18 years)
Intervention: treatment	Liver resection	
	Adjuvant and neoadjuvant treatment (including radio- and/or chemotherapy)	
Comparators: control	Liver resection with neoadjuvant treatment vs liver resection alone	
	Liver resection with adjuvant treatment vs liver resection alone	
	Liver resection with neoadjuvant and adjuvant treatment vs liver resection alone	
Outcomes	Primary outcome: OS	Studies not reporting the OS
	Secondary outcomes: tumor-free survival, quality of life, increase in R0/R1 resectability	
Study design	Randomized controlled trials	Case reports
	Prospective and retrospective comparative cohort studies	
	Case-control studies	
	Case series	

**Table 3:** Eligibility criteria for review on liver transplantation [35].

Study characteristics	Inclusion criteria	Exclusion criteria
Patient population	Patients with nonresectable neuroendocrine tumor (NET) liver metastases	Children or adolescents (under the age of 18 years)
	Patients who underwent liver transplantation or palliative liver resection or nonsurgical treatment (PRRT, chemotherapy, biotherapy)	
Intervention: treatment	Liver transplantation (orthotopic, deceased donor liver transplantation, multivisceral transplantation, living-donor liver transplantation)	
	Palliative liver resection	
	Nonsurgical treatment (chemotherapy, biotherapy, locally ablative techniques, radionuclide therapy)	
	Delay of liver transplantation	
	Living-donor liver donation	
	Deceased donor liver donation	
Intervention: comparison	Liver transplantation vs palliative liver resection vs nonsurgical treatment (chemotherapy, biotherapy, locally ablative techniques, radionuclide therapy)	
	Early vs late transplantation	
Outcomes	Primary outcome: OS	Studies that do not report the OS
	Secondary outcomes: progression free survival, quality of life	
Study design	Randomized controlled trials	Case reports
	Prospective and retrospective comparative cohort studies	
	Case-control studies	
	Case series	

**Table 4:** Eligibility criteria for review on resection of the primary tumor [36].

Study characteristics	Inclusion criteria	Exclusion criteria
Patient population	Patients with neuroendocrine tumors and nonresectable liver metastases	Children or adolescents (under the age of 18 years)
	Primary tumor located in pancreas, intestine, or lung	
	Patients with neuroendocrine tumors and nonresectable liver metastases who underwent resection or nonsurgical treatment of the primary	
Intervention: treatment	Resection of the primary tumor	
	PRRT	
	Chemotherapy	
	Biotherapy	
Comparators: control	Patients with neuroendocrine tumors and nonresectable liver metastases who received resection of the primary vs nonsurgical treatment of the primary tumor	
Outcomes	Primary outcome: OS	Studies that do not report the OS
	Secondary outcome: progression-free survival, quality of life	
Study design	Randomized controlled trials	Case reports
	Prospective and retrospective comparative cohort studies	
	Case-control studies	
	Case series	

### Search

Librarians of the Medical Library Careum, University of Zurich, Switzerland, developed the electronic search strategy to query databases and to identify all potentially relevant articles (see Multimedia Appendix 1). The following databases were searched: Medical Literature Analysis and Retrieval System Online, Excerpta Medica Database, and the Cochrane Library (Cochrane Database of Systematic Reviews, Database of Abstracts of Reviews of Effects, and Cochrane Central Register of Controlled Trials). The investigators were provided with an endnote file containing all identified titles and, if available, the corresponding abstracts. Additional articles were retrieved through manual search or scanning of reference lists. Titles and/or abstracts of all identified records were independently screened by 2 members of the review team to ascertain their relevance and to identify studies that potentially meet the inclusion criteria as outlined in Tables 1-4. The full text of each of these potentially relevant studies was then assessed for eligibility. Any disagreement was resolved through discussion with a third review team member. A predefined protocol was used to extract data from the included studies for the assessment of study quality and evidence synthesis.

### Data Extraction

The following parameters will be chosen for data extraction: first author’s name, publication year, answering scientific questions, study design, total number of patients, number of patients in the study group, number of patients in the comparison group, type of nonsurgical treatment, age (mean, SD, median), male-to-female ratio, progression-free survival, OS, quality of life (tools), and hazard risk ratio. The Grading of Recommendations Assessment, Development, and Evaluation will be used to grade the quality (level) of evidence and the strength of recommendations [37].

A narrative synthesis of the findings from the included studies will be provided. A quantitative synthesis will be used for studies that are sufficiently homogenous from a clinical (population comparability, interventions, and outcomes) and from a statistical perspective (heterogeneity, eg, I^2^<50%). It is anticipated that there will be a limited scope for meta-analysis despite a relatively large number of studies due to different outcome measurements of the existing trials as such tumors are rare. However, results from studies using the same type of intervention and comparator with the same outcome measurements will be pooled using a random-effects meta-analysis. In addition, risk ratios for binary outcomes, 95% CI, and two-sided *P* values will be calculated for each outcome.

## Results

This study is ongoing and presents a protocol system of four systematic reviews that will assist in determining the effectiveness of liver resection versus nonsurgical treatment of patients with NET liver metastases. This study is also assumed to investigate the impact of neoadjuvant and adjuvant treatment options on the tumor-free survival, the role of liver transplantation, and the relevance of primary tumor resection in presence of unresectable liver metastasis.

## Discussion

The use of surgical strategies for the treatment of patients with liver metastases from NET is still controversial. An important step toward developing a consensus is to summarize the existing scientific literature.

Regarding liver resection in patients with liver metastases from NETs, Gurusamy et al presented 2 Cochrane Collaboration systematic reviews on liver resection and cytoreductive surgery versus nonsurgical treatments in patients with resectable and nonresectable liver metastases. Publications until July 2008 were included in their reviews. Based on nonrandomized studies, they came to the conclusion that liver resection “appears to be the main stay curative treatment for neuroendocrine liver metastases” [38,39]. Our systematic review will consider data published until 2012.

Regarding neoadjuvant therapies, PRRT seems to be a possible neoadjuvant option in initially unresectable primary NETs, while its benefit in the treatment of NET liver metastases needs to be elucidated [40]. Apart from PRRT, chemotherapy and biologic therapies (eg, octreotide) also need to be evaluated in the neoadjuvant and adjuvant settings.

Liver transplantation is a controversially discussed treatment option in patients with liver metastases from NETs, because it is not clear which patients benefit most from this therapeutic strategy. Máthé et al performed a systematic review to investigate the benefit of liver transplantation for hepatic metastases of pancreatic NETs and grouped patients according to their age (less than 55 years or 55 years or older) and surgical procedure they underwent (pancreatic resection prior to liver transplantation or simultaneous resection). The 5-year OS was found to be significantly different between patients who were less than 55 years of age and had pancreatic resection prior to transplantation compared to patients who were 55 years of age or older and underwent simultaneous resection (5-year OS 61% vs 0%) [41]. Reaching an overall 5-year survival of incredibly 96%, the Milan criteria seem to provide a good foundation for further improvement of the selection criteria [16]. Therefore, and in combination with the scarcity of donor organs, it is crucial to evaluate and define accurate selection criteria for potential transplant recipients to offer these patients the most promising and evidence-based treatment.

Surgical resection of NETs is the treatment strategy whenever a curative intent is anticipated. However, it is not clear whether resection of the primary NET is still beneficial in advanced disease stage presenting with unresectable liver metastases. Bettini et al investigated the role of primary tumor resection in nonfunctioning pancreatic NETs with unresectable liver metastases [42]. OS did not differ significantly, although survival was longer in patients with resected primary tumor. A significant difference in improvement of symptoms in favor of primary resection was observed, although quality of life was not assessed objectively. Therefore, resection was considered as palliative therapy in order to relief symptoms related to primary tumor mass and prevent obstructive complications such as bleeding, acute pancreatitis, or jaundice.

The four systematic reviews described in this protocol will help to elucidate the role of surgical strategies and serve as a basis for developing clinical practice guidelines.

## Multimedia Appendix 1

Results of literature search from Medical Literature Analysis and Retrieval System Online, Excerpta Medica Database, and the Cochrane Library.

## Multimedia Appendix 2

CONSORT-EHEALTH-checklist V1.6.2 [43].

## Abbreviations

NET: neuroendocrine tumor
OS: overall survival
PRRT: peptide radio receptor therapy
RCT: randomized controlled trial
SIRT: selective internal radiation therapy
TACE: transcatheter arterial chemoembolization
TAE: transcatheter arterial embolization
