# Supplementary material for: Transplantation and Surgical Strategies in Patients With Neuroendocrine Liver Metastases: Protocol of Four Systematic Reviews
Source: JMIR Res Protoc. 2013 Dec 23;2(2):e58. doi: 10.2196/resprot.2891 (PMC3875902; doi:10.2196/resprot.2891)
Supplement: Supplementary file 1 [file resprot_v2i2e58_app1.pdf]

Results of Literature Search

NET and Liver Metastases – Session 8

Search Protocols:
Session_8_Ovid_ Search Results.pdf
Session_8_EMBASE.pdf
Session_8_Cochrane.docx

	Time span	References	References after Deduplication
**Medline/Premedline**	no limit	602	595
**Embase**	no limit	980	515
**Cochrane**	no limit	11	1
**Pool**		1593	1111

References in *Session_8.enlx*

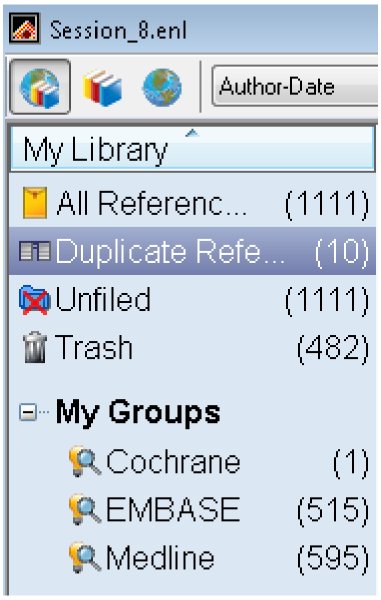

Database(s): **Ovid MEDLINE(R), Ovid MEDLINE(R) In-Process & Other Non-Indexed Citations, Ovid MEDLINE(R) Daily and Ovid OLDMEDLINE(R)** 1946 to Present

Search Strategy:

#	Searches	Results
1	exp Neuroendocrine Tumors/ not (exp melanoma/ or exp neurilemmoma/)	39296
2	exp Apudoma/	443
3	exp Carcinoid Tumor/	10844
4	exp Adenoma, Islet Cell/	7319
5	exp Insulinoma/	3679
6	exp Carcinoma, Islet Cell/	2146
7	exp Gastrinoma/	838
8	exp Glucagonoma/	665
9	exp Somatostatinoma/	283
10	exp Vipoma/	412
11	exp Multiple Endocrine Neoplasia/	4325
12	exp Pancreatic Neoplasms/	49120
13	or/1-12	87853
14	((neuroendocrine or gastroenteropancreatic) adj3 (tumo?r$ or neoplasm or adenoma$ or carcinoma$)).ti,ab.	8089
15	(carcinoid$ or insulinoma$ or gastrinoma$ or glucagonoma$ or vipoma$ or somatostatinoma$ or apudoma$ or adenoma$ or nesidioblastoma$ or argentaffinoma$).ti,ab.	78242
16	(multiple endocrine adj3 (neoplasia$ or adenopath$ or adenomatos?s or neoplasm$)).ti,ab.	4319
17	((islet or island or beta) adj3 (cell adj2 (tumo?r$ or adenoma$ or carcinoma$))).ti,ab.	2655
18	(familial endocrine adj3 adenomatos?s).ti,ab.	8
19	((diarrheogenic or VIP secreting) adj3 tumo?r$).ti,ab.	39
20	watery diarrh$ syndrome.ti,ab.	58
21	"pancreatic cholera".ti,ab.	59
22	((verner morrison or zollinger ellison) adj3 syndrome).ti,ab.	2085
23	or/14-22	89360
24	13 or 23	152055
25	exp Liver Neoplasms/sc [Secondary]	23515
26	exp Neoplasm Metastasis/	141543
27	exp Liver/	366332

28	(liver or hepatic).ti,ab.	649227
29	27 or 28	759333
30	26 and 29	10559
31	((secondar$ or spread or advanced) adj3 (tumo?r$ or cancer or neoplasm$ or adenoma$ or carcinoma$)).ti,ab.	66963
32	((tumo?r$ or cancer or neoplasm$ or adenoma$ or carcinoma$) adj10 (liver or hepatic)).ti,ab.	68275
33	31 and 32	3529
34	((liver or hepatic) adj3 metasta*).ti,ab.	23489
35	25 or 30 or 33 or 34	40318
36	24 and 35	6008
37	limit 36 to animals	514
38	limit 37 to humans	318
39	37 not 38	196
40	36 not 39	5812
41	limit 40 to "all child (0 to 18 years)"	426
42	limit 41 to "all adult (19 plus years)"	349
43	41 not 42	77
44	40 not 43	5735
45	exp Hepatectomy/	19362
46	exp Liver Neoplasms/sc and exp Liver Neoplasms/su [Secondary,Surgery]	5189
47	((resection or segmentectomy or metastasectomy or surgery) adj5 (liver or hepatic)).ti,ab.	15934
48	45 or 46 or 47	30292
49	ethanol injection.ti,ab.	1729
50	(ablative adj3 therap*).ti,ab.	1307
51	(cryosurg* or cryoablat* or radioablat* or thermoablat* or microwave).ti,ab.	22209
52	((radiofrequency or radio-frequency or RF) adj3 ablat*).ti,ab.	10721
53	(biotherapy or emboli?ation or chemoemboli?ation or radioemboli?ation or chemo-emboli?ation or radio-emboli?ation).ti,ab.	32355
54	pprt.ti,ab.	11
55	(selective adj3 (radionuclide or radiation)).ti,ab.	357
56	((radionuclide or radiation) adj3 (treatment or therapy)).ti,ab.	55050
57	55 and 56	194
58	exp Catheter Ablation/ or exp Ablation Techniques/	80694
59	exp Cryosurgery/	10363
60	exp Chemoembolization, Therapeutic/	2897

61	49 or 50 or 51 or 52 or 53 or 54 or 57 or 58 or 60	133558
62	48 or 61	160170
63	44 and 62	1308
64	exp Disease-Free Survival/ or exp Survival Analysis/ or exp Survival/ or exp Survival Rate/	247642
65	exp "Quality of Life"/	99085
66	(quality adj3 life).ti,ab.	121923
67	surviv*.mp. [mp=title, abstract, original title, name of substance word, subject heading word, protocol supplementary concept, rare disease supplementary concept, unique identifier]	798042
68	or/64-67	950290
69	63 and 68	602

501. **Surgical resection of metastatic liver tumors. [Review] [69 refs]**

Kavolius J. Fong Y. Blumgart LH.

Surgical Oncology Clinics of North America. 5(2):337-52, 1996 Apr.
[Journal Article. Review]

UI: 9019355

Authors Full Name

Kavolius, J. Fong, Y. Blumgart, L H.

embase BIOMEDICAL ANSWERS

Embase Session Results

No.	Query	Results
**#24**	**#18** AND **#23**	**980**
**#23**	**#19** OR **#20** OR **#21** OR **#22**	**1,086,668**
**#22**	**surviv***:ab,ti	**764,334**
**#21**	(**quality** NEAR/3 **life**):ab,ti	**167,597**
**#20**	**'quality of life'**/exp	**204,915**
**#19**	**'survival'**/exp	**454,858**
**#18**	**#1** AND **#17**	**1,962**
**#17**	**#5** OR **#16**	**146,936**
**#16**	**#6** OR **#7** OR **#8** OR **#9** OR **#10** OR **#11** OR **#14** OR **#15**	**111,176**
**#15**	**'radiofrequency ablation'**/exp OR **'catheter ablation'**/exp OR **'chemoembolization'**/exp OR **'cryosurgery'**/exp	**41,866**
**#14**	**#12** AND **#13**	**273**
**#13**	((**radionuclide** OR **radiation**) NEAR/3 (**treatment** OR **therapy**)):ab,ti	**69,818**
**#12**	(**selective** NEAR/3 (**radionuclide** OR **radiation**)):ab,ti	**462**
**#11**	**pprt**:ab,ti	**20**
**#10**	**biotherapy**:ab,ti OR **emboli?ation**:ab,ti OR **chemoemboli?ation**:ab,ti OR **radioemboli?ation**:ab,ti OR **'chemo emboli?ation'**:ab,ti OR **'radio emboli?ation'**:ab,ti	**48,194**
**#9**	((**radiofrequency** OR **'radio frequency'** OR **rf**) NEAR/3 **ablat***):ab,ti	**14,858**
**#8**	**cryosurg***:ab,ti OR **cryoablat***:ab,ti OR **radioablat***:ab,ti OR **thermoablat***:ab,ti OR **microwave**:ab,ti	**27,553**
**#7**	(**ablative** NEAR/3 **therap***):ab,ti	**1,748**
**#6**	(**ethanol** NEAR/3 **injection**):ab,ti	**3,025**
**#5**	**#2** OR **#3** OR **#4**	**40,834**
**#4**	((**resection** OR **segmentectomy** OR **metastasectomy** OR **surgery**) NEAR/5 (**liver** OR **hepatic**)):ab,ti	**20,380**
**#3**	**'liver metastasis'**/exp/dm_su	**5,080**

**#2**	**'liver resection'**/exp	**30,026**
**#1**		

**#1.43**	**#1.39** NOT **#1.42**	**7,288**
**#1.42**	**#1.40** NOT **#1.41**	
**#1.41**	**#1.35** NOT **#1.38** AND ([newborn]/lim OR [infant]/lim OR [preschool]/lim OR [school]/lim OR [child]/lim OR [adolescent]/lim) AND ([adult]/lim OR [aged]/lim)	
**#1.40**	**#1.35** NOT **#1.38** AND ([newborn]/lim OR [infant]/lim OR [preschool]/lim OR [school]/lim OR [child]/lim OR [adolescent]/lim)	
**#1.39**	**#1.35** NOT **#1.38**	
**#1.38**	**#1.36** NOT **#1.37**	
**#1.37**	**#1.28** AND **#1.34** AND [animals]/lim AND [humans]/lim	
**#1.36**	**#1.28** AND **#1.34** AND [animals]/lim	
**#1.35**	**#1.28** AND **#1.34**	
**#1.34**	**#1.29** OR **#1.32** OR **#1.33**	
**#1.33**	(**metasta*** NEAR/3 (**liver** OR **hepatic**)):ab,ti	
**#1.32**	**#1.30** AND **#1.31**	
**#1.31**	((**tumor*** OR **tumour*** OR **cancer** OR **neoplasm*** OR **adenoma*** OR **carcinoma***) NEAR/10 (**liver** OR **hepatic**)):ab,ti	
**#1.30**	((**secondar*** OR **spread** OR **advanced**) NEAR/3 (**tumor*** OR **tumour*** OR **cancer** OR **neoplasm*** OR **adenoma*** OR **carcinoma***)):ab,ti	
**#1.29**	**'liver metastasis'**/exp	
**#1.28**	**#1.13** OR **#1.27**	
**#1.27**	**#1.14** OR **#1.15** OR **#1.16** OR **#1.19** OR **#1.20** OR **#1.21** OR **#1.24** OR **#1.25** OR **#1.26**	
**#1.26**	((**'verner morrison'** OR **'zollinger ellison'**) NEAR/3 **syndrome**):ab,ti	
**#1.25**	**'pancreatic cholera'**:ab,ti	
**#1.24**	**#1.22** AND **#1.23**	
**#1.23**	(**diarr*** NEAR/3 **syndrome**):ab,ti	
**#1.22**	(**watery** NEAR/3 **diarr***):ab,ti	
**#1.21**	((**diarrheogenic** OR **'vip secreting'** OR **'vip secreting'**) NEAR/3 (**tumor*** OR **tumour***)):ab,ti	
**#1.20**	**familial**:ab,ti AND (**endocrine** NEAR/3 **adenomatos?s**):ab,ti	
**#1.19**	**#1.17** AND **#1.18**	
**#1.18**	((**islet** OR **island** OR **beta**) NEAR/3 **cell**):ab,ti	
**#1.17**	(**cell** NEAR/2 (**tumor*** OR **tumour*** OR **adenoma*** OR **carcinoma***)):ab,ti	
**#1.16**	**multiple**:ab,ti AND (**endocrine** NEAR/3 (**neoplasia*** OR **adenopath*** OR	

	**adenomatos?s** OR **neoplasm***)):ab,ti
**#1.15**	**carcinoid***:ab,ti OR **insulinoma***:ab,ti OR **gastrinoma***:ab,ti OR **glucagonoma***:ab,ti OR **vipoma***:ab,ti OR **somatostatinoma***:ab,ti OR **apudoma***:ab,ti OR **adenoma***:ab,ti OR **nesidioblastoma***:ab,ti OR **argentaffinoma***:ab,ti
**#1.14**	((**neuroendocrine** OR **gastroenteropancreatic**) NEAR/3 (**tumor*** OR **tumour*** OR **neoplasm** OR **adenoma*** OR **carcinoma***)):ab,ti
**#1.13**	**#1.1** OR **#1.2** OR **#1.3** OR **#1.4** OR **#1.5** OR **#1.6** OR **#1.7** OR **#1.8** OR **#1.9** OR **#1.10** OR **#1.11** OR **#1.12**
**#1.12**	**'pancreas tumor'**/exp
**#1.11**	**'multiple endocrine neoplasia'**/exp
**#1.10**	**'vipoma'**/exp
**#1.9**	**'somatostatinoma'**/exp
**#1.8**	**'glucagonoma'**/exp
**#1.7**	**'gastrinoma'**/exp
**#1.6**	**'pancreas islet cell carcinoma'**/exp
**#1.5**	**'insulinoma'**/exp
**#1.4**	**'pancreas islet cell tumor'**/exp
**#1.3**	**'carcinoid'**/exp
**#1.2**	**'apudoma'**/exp
**#1.1**	**'neuroendocrine tumor'**/exp

ELSEVIER Copyright © 2012 Elsevier B.V. All rights reserved. Embase is a trademark of Elsevier B.V.

Search Name: Net Session_8

Comments: Breitenstein USZ

Save Date: 2012-05-23 09:49:13.123

ID	Search
#1	MeSH descriptor Neuroendocrine Tumors explode all trees
#2	MeSH descriptor Apudoma explode all trees
#3	MeSH descriptor Carcinoid Tumor explode all trees
#4	MeSH descriptor Adenoma, Islet Cell explode all trees
#5	MeSH descriptor Insulinoma explode all trees
#6	MeSH descriptor Carcinoma, Islet Cell explode all trees
#7	MeSH descriptor Gastrinoma explode all trees
#8	MeSH descriptor Glucagonoma explode all trees
#9	MeSH descriptor Somatostatinoma explode all trees
#10	MeSH descriptor Vipoma explode all trees
#11	MeSH descriptor Multiple Endocrine Neoplasia explode all trees
#12	MeSH descriptor Pancreatic Neoplasms explode all trees
#13	(#1 OR #2 OR #3 OR #4 OR #5 OR #6 OR #7 OR #8 OR #9 OR (#10 AND #11) OR #12)
#14	((neuroendocrine OR gastroenteropancreatic) NEAR/3 (tumor* OR tumour* OR neoplasm OR adenoma* OR carcinoma*)):ti,ab,kw or (carcinoid* or insulinoma* or gastrinoma* or glucagonoma* or vipoma* or somatostatinoma* or apudoma* or adenoma* or nesidioblastoma* or argentaffinoma*):ti,ab,kw or ((multiple endocrine) NEAR/3 (neoplasia* OR adenopath* OR adenomatos?s OR neoplasm*)):ti,ab,kw
#15	((islet or island or beta) NEAR/3 (cell NEAR/2 (tumo?r* or adenoma* or carcinoma*))):ti,ab,kw
#16	((familial endocrine) NEAR/3 adenomatos?s):ti,ab,kw
#17	((diarrheogenic OR 'vip secreting' OR 'vip secreting') NEAR/3 (tumor* OR tumour*)):ti,ab,kw
#18	(watery diarrh$ syndrome):ti,ab,kw
#19	"pancreatic cholera":ti,ab,kw
#20	(('verner morrison' OR 'zollinger ellison') NEAR/3 syndrome):ti,ab,kw
#21	(#14 OR #15)
#22	(#13 OR #21)
#23	MeSH descriptor Neoplasm Metastasis explode all trees
#24	MeSH descriptor Liver explode all trees
#25	(liver or hepatic):ti or (liver or hepatic):ab
#26	(#24 OR #25)
#27	(#23 AND #26)
#28	((secondar* OR spread OR advanced) NEAR/3 (tumor* OR tumour* OR cancer OR neoplasm* OR adenoma* carcinoma*)):ti,ab,kw

#29 ((tumor* OR tumour* OR cancer OR neoplasm* OR adenoma* OR carcinoma*) NEAR/10 (liver OR hepatic)):ti,ab,kw

#30 (#28 AND #29)

#31 (metasta* NEAR/3 (liver OR hepatic)):ti,ab,kw

#32 MeSH descriptor Liver Neoplasms explode all trees with qualifier: SC

#33 (#27 OR #30 OR #31 OR #32)

#34 (#22 AND #33)

#35 MeSH descriptor Melanoma explode all trees

#36 MeSH descriptor Neurilemma explode all trees

#37 (#36 OR #35)

#38 (#34 AND NOT #37)

#39 MeSH descriptor Hepatectomy explode all trees

#40 MeSH descriptor Liver Neoplasms explode all trees with qualifier: SC

#41 MeSH descriptor Liver Neoplasms explode all trees with qualifier: SU

#42 (#40 AND #41)

#43 MeSH descriptor Cryosurgery explode all trees

#44 ((resection OR segmentectomy OR metastasectomy OR surgery) NEAR/5 (liver OR hepatic)):ti,ab,kw

#45 (#39 OR #42 OR #43 OR #44)

#46 (ethanol NEAR/3 injection):ti,ab,kw or (ablative NEAR/3 therap*):ti,ab,kw

#47 (crosurger* or cryoablat* or radioablat* or thermoablat*):ti,ab,kw or ((radiofrequency OR 'radio frequency' OR rf) NEAR/3 ablat*):ti,ab,kw or (biotherapy or emboli?ation or chemoemboli?ation or radioemboli?ation or chemo-emboli?ation or radio-emboli?ation):ti,ab,kw or (pprt):ti,ab,kw

#48 (selective NEAR/3 (radionuclide OR radiation)):ti,ab,kw and ((radionuclide OR radiation) NEAR/3 (treatment OR therapy)):ti,ab,kw

#49 MeSH descriptor Catheter Ablation explode all trees

#50 MeSH descriptor Ablation Techniques explode all trees

#51 MeSH descriptor Chemoembolization, Therapeutic explode all trees

#52 (#46 OR #47 OR #48 OR #49 OR #50 OR #51)

#53 (#45 OR #52)

#54 (#38 AND #53)

#55 MeSH descriptor Survival Rate explode all trees

#56 MeSH descriptor Survival Analysis explode all trees

#57 MeSH descriptor Disease-Free Survival explode all trees

#58 MeSH descriptor Quality of Life explode all trees

#59 (quality NEAR/3 life):ti,ab,kw or (surviv*):ti,ab,kw

#60 (#55 OR #56 OR #57 OR #58 OR #59)

#61 (#54 AND #60)

ID	Search	Hits	Edit	Delete
#1	MeSH descriptor **Neuroendocrine Tumors** explode all trees	1156	edit	delete
#2	MeSH descriptor **Apudoma** explode all trees	0	edit	delete
#3	MeSH descriptor **Carcinoid Tumor** explode all trees	44	edit	delete
#4	MeSH descriptor **Adenoma, Islet Cell** explode all trees	15	edit	delete
#5	MeSH descriptor **Insulinoma** explode all trees	9	edit	delete
#6	MeSH descriptor **Carcinoma, Islet Cell** explode all trees	6	edit	delete
#7	MeSH descriptor **Gastrinoma** explode all trees	3	edit	delete
#8	MeSH descriptor **Glucagonoma** explode all trees	0	edit	delete
#9	MeSH descriptor **Somatostatinoma** explode all trees	1	edit	delete
#10	MeSH descriptor **Vipoma** explode all trees	2	edit	delete
#11	MeSH descriptor **Multiple Endocrine Neoplasia** explode all trees	7	edit	delete
#12	MeSH descriptor **Pancreatic Neoplasms** explode all trees	690	edit	delete
#13	(#1 OR #2 OR #3 OR #4 OR #5 OR #6 OR #7 OR #8 OR #9 OR (#10 AND #11) OR #12)	1822	edit	delete
#14	((neuroendocrine OR gastroenteropancreatic) NEAR/3 (tumor* OR tumour* OR neoplasm OR adenoma* OR carcinoma*)):ti,ab,kw or (carcinoid* or insulinoma* or gastrinoma* or glucagonoma* or vipoma* or somatostatinoma* or apudoma* or adenoma* or nesidioblastoma* or argentaffinoma*):ti,ab,kw or ((multiple endocrine) NEAR/3 (neoplasia* OR adenopath* OR adenomatos?s OR neoplasm*)):ti,ab,kw	1304	edit	delete
#15	((islet or island or beta) NEAR/3 (cell NEAR/2 (tumo?r* or adenoma* or carcinoma*))):ti,ab,kw	15	edit	delete
#16	((familial endocrine) NEAR/3 adenomatos?s):ti,ab,kw	0	edit	delete
#17	((diarrheogenic OR 'vip secreting' OR 'vip secreting') NEAR/3 (tumor* OR tumour*)):ti,ab,kw	0	edit	delete
#18	(watery diarrh$ syndrome):ti,ab,kw	0	edit	delete
#19	"pancreatic cholera":ti,ab,kw	0	edit	delete
#20	(('verner morrison' OR 'zollinger ellison') NEAR/3 syndrome):ti,ab,kw	0	edit	delete
#21	(#14 OR #15)	1310	edit	delete
#22	(#13 OR #21)	3010	edit	delete
#23	MeSH descriptor **Neoplasm Metastasis** explode all trees	3275	edit	delete
#24	MeSH descriptor **Liver** explode all trees	2339	edit	delete
#25	(liver or hepatic):ti or (liver or hepatic):ab	24742	edit	delete
#26	(#24 OR #25)	25211	edit	delete
#27	(#23 AND #26)	185	edit	delete
#28	((secondar* OR spread OR advanced) NEAR/3 (tumor* OR tumour* OR cancer OR neoplasm* OR adenoma* carcinoma*)):ti,ab,kw	7713	edit	delete
#29	((tumor* OR tumour* OR cancer OR neoplasm* OR adenoma* OR carcinoma*) NEAR/10 (liver OR hepatic)):ti,ab,kw	2779	edit	delete
#30	(#28 AND #29)	612	edit	delete
#31	(metasta* NEAR/3 (liver OR hepatic)):ti,ab,kw	791	edit	delete
#32	MeSH descriptor **Liver Neoplasms** explode all trees with qualifier: **SC**	530	edit	delete
#33	(#27 OR #30 OR #31 OR #32)	1219	edit	delete
#34	(#22 AND #33)	101	edit	delete
#35	MeSH descriptor **Melanoma** explode all trees	952	edit	delete
#36	MeSH descriptor **Neurilemma** explode all trees	1	edit	delete
#37	(#36 OR #35)	954	edit	delete
#38	(#34 AND NOT #37)	69	edit	delete
#39	MeSH descriptor **Hepatectomy** explode all trees	404	edit	delete
#40	MeSH descriptor **Liver Neoplasms** explode all trees with qualifier: **SC**	530	edit	delete
#41	MeSH descriptor **Liver Neoplasms** explode all trees with qualifier: **SU**	410	edit	delete
#42	(**#40** AND **#41**)	99	edit	delete
#43	MeSH descriptor **Cryosurgery** explode all trees	253	edit	delete
#44	((resection OR segmentectomy OR metastasectomy OR surgery) NEAR/5 (liver OR hepatic)):ti,ab,kw	1345	edit	delete
#45	(#39 OR #42 OR #43 OR #44)	1659	edit	delete
#46	(ethanol NEAR/3 injection):ti,ab,kw or (ablative NEAR/3 therap*):ti,ab,kw	176	edit	delete
#47	(crosurger* or cryoablat* or radioablat* or thermoablat*):ti,ab,kw or ((radiofrequency OR 'radio frequency' OR rf) NEAR/3 ablat*):ti,ab,kw or (biotherapy or emboli?ation or chemoemboli?ation or radioemboli?ation or chemo-emboli?ation or radio-emboli?ation):ti,ab,kw or (pprt):ti,ab,kw	1504	edit	delete
#48	(selective NEAR/3 (radionuclide OR radiation)):ti,ab,kw and ((radionuclide OR radiation) NEAR/3 (treatment OR therapy)):ti,ab,kw	10	edit	delete
#49	MeSH descriptor **Catheter Ablation** explode all trees	915	edit	delete

#50	MeSH descriptor **Ablation Techniques** explode all trees	4101	edit	delete
#51	MeSH descriptor **Chemoembolization, Therapeutic** explode all trees	192	edit	delete
#52	(#46 OR #47 OR #48 OR #49 OR #50 OR #51)	5267	edit	delete
#53	(#45 OR #52)	6515	edit	delete
#54	(#38 AND #53)	18	edit	delete
#55	MeSH descriptor **Survival Rate** explode all trees	7198	edit	delete
#56	MeSH descriptor **Survival Analysis** explode all trees	12895	edit	delete
#57	MeSH descriptor **Disease-Free Survival** explode all trees	3635	edit	delete
#58	MeSH descriptor **Quality of Life** explode all trees	11889	edit	delete
#59	(quality NEAR/3 life):ti,ab,kw or (surviv*):ti,ab,kw	56737	edit	delete
#60	(#55 OR #56 OR #57 OR #58 OR #59)	58558	edit	delete
#61	(#54 AND #60)	11	edit	delete

Results of Literature Search

NET and Liver Metastases – Session 9

Search Protocols:
Session_9_Ovid_ Search Results.pdf
Session_9_EMBASE.pdf
Session_9_Cochrane.docx

	Time span	References	References after Deduplication
**Medline/Premedline**	no limit	777	770
**Embase**	no limit	1116	615
**Cochrane**	no limit	16	0
**Pool**		1909	1385

References in *Session_9.enlx*

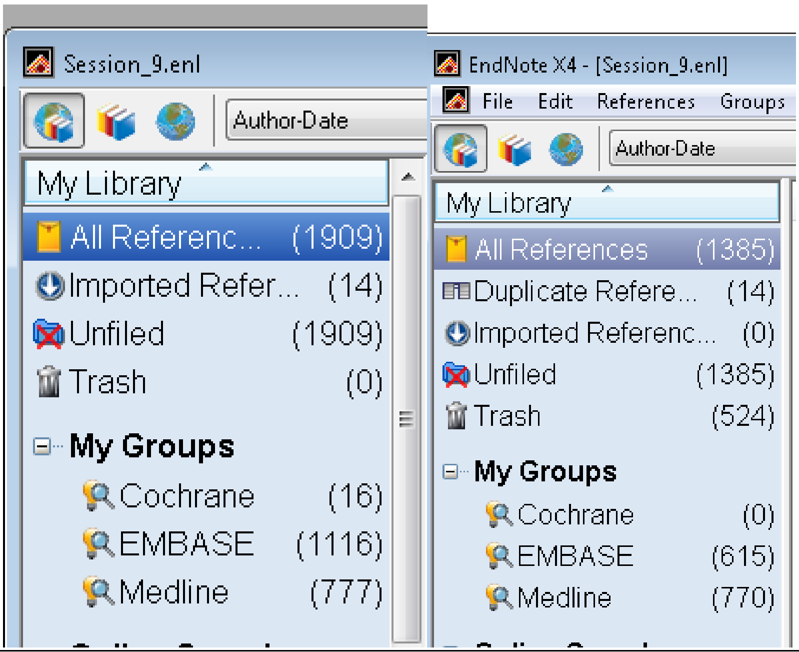

Database(s): **Ovid MEDLINE(R), Ovid MEDLINE(R) In-Process & Other Non-Indexed Citations, Ovid MEDLINE(R) Daily and Ovid OLDMEDLINE(R)** 1946 to Present

Search Strategy:

#	Searches	Results
1	exp Neuroendocrine Tumors/ not (exp melanoma/ or exp neurilemmoma/)	39308
2	exp Apudoma/	443
3	exp Carcinoid Tumor/	10848
4	exp Adenoma, Islet Cell/	7321
5	exp Insulinoma/	3681
6	exp Carcinoma, Islet Cell/	2146
7	exp Gastrinoma/	838
8	exp Glucagonoma/	665
9	exp Somatostatinoma/	283
10	exp Vipoma/	412
11	exp Multiple Endocrine Neoplasia/	4327
12	exp Pancreatic Neoplasms/	49169
13	or/1-12	87915
14	((neuroendocrine or gastroenteropancreatic) adj3 (tumo?r$ or neoplasm or adenoma$ or carcinoma$)).ti,ab.	8099
15	(carcinoid$ or insulinoma$ or gastrinoma$ or glucagonoma$ or vipoma$ or somatostatinoma$ or apudoma$ or adenoma$ or nesidioblastoma$ or argentaffinoma$).ti,ab.	78273
16	(multiple endocrine adj3 (neoplasia$ or adenopath$ or adenomatos?s or neoplasm$)).ti,ab.	4346
17	((islet or island or beta) adj3 (cell adj2 (tumo?r$ or adenoma$ or carcinoma$))).ti,ab.	2655
18	(familial endocrine adj3 adenomatos?s).ti,ab.	8
19	((diarrheogenic or VIP secreting) adj3 tumo?r$).ti,ab.	39
20	watery diarrh$ syndrome.ti,ab.	58
21	"pancreatic cholera".ti,ab.	59
22	((verner morrison or zollinger ellison) adj3 syndrome).ti,ab.	2086
23	or/14-22	89417
24	13 or 23	152163
25	exp Liver Neoplasms/sc [Secondary]	23534
26	exp Neoplasm Metastasis/	141648
27	exp Liver/	366430

28	(liver or hepatic).ti,ab.	649222
29	27 or 28	759339
30	26 and 29	10571
31	((secondar$ or spread or advanced) adj3 (tumo?r$ or cancer or neoplasm$ or adenoma$ or carcinoma$)).ti,ab.	66995
32	((tumo?r$ or cancer or neoplasm$ or adenoma$ or carcinoma$) adj10 (liver or hepatic)).ti,ab.	68286
33	31 and 32	3532
34	((liver or hepatic) adj3 metasta*).ti,ab.	23506
35	25 or 30 or 33 or 34	40344
36	24 and 35	6016
37	limit 36 to animals	515
38	limit 37 to humans	319
39	37 not 38	196
40	36 not 39	5820
41	limit 40 to "all child (0 to 18 years)"	426
42	limit 41 to "all adult (19 plus years)"	349
43	41 not 42	77
44	40 not 43	5743
45	(pancreatectomy or pancreaticoduodenectomy or pulmonectomy or lobectomy or colectomy or ileocolectomy or pneumonectomy).mp. [mp=title, abstract, original title, name of substance word, subject heading word, protocol supplementary concept, rare disease supplementary concept, unique identifier]	57869
46	exp Pancreatectomy/	8525
47	exp Pancreaticoduodenectomy/	3925
48	exp Pneumonectomy/	19289
49	exp Colectomy/	13419
50	exp Ileum/su [Surgery]	8904
51	exp Pancreas/su [Surgery]	4369
52	exp Lung/su [Surgery]	5959
53	exp Intestines/su [Surgery]	45153
54	exp Pancreatic Neoplasms/su [Surgery]	10705
55	exp Lung Neoplasms/su [Surgery]	21220
56	exp Intestinal Neoplasms/su [Surgery]	34158
57	or/45-56	146678
58	ethanol injection.ti,ab.	1730

59	(ablative adj3 therap*).ti,ab.	1308
60	(cryosurg* or cryoablat* or radioablat* or thermoablat*).ti,ab.	5347
61	((radiofrequency or radio-frequency or RF) adj3 ablat*).ti,ab.	10740
62	(biotherapy or emboli?ation or chemoemboli?ation or radioemboli?ation).ti,ab.	32370
63	pprt.ti,ab.	11
64	(selective adj3 (radionuclide or radiation)).ti,ab.	358
65	((radionuclide or radiation) adj3 (treatment or therapy)).ti,ab.	55058
66	64 and 65	195
67	exp Catheter Ablation/ or exp Ablation Techniques/	80752
68	exp Cryosurgery/	10368
69	exp Chemoembolization, Therapeutic/	2905
70	58 or 59 or 60 or 61 or 62 or 63 or 66 or 67 or 68 or 69	117553
71	57 or 70	260834
72	44 and 71	1801
73	exp Disease-Free Survival/ or exp Survival Analysis/ or exp Survival/ or exp Survival Rate/	247981
74	exp "Quality of Life"/	99185
75	(quality adj3 life).ti,ab.	121996
76	surviv*.mp.	798316
77	or/73-76	950694
78	72 and 77	777

501. **Unusually aggressive rectal carcinoid metastasizing to larynx, pancreas, adrenal glands, and brain.**

Danikas D. Theodorou SJ. Matthews WE. Rienzo AA.

American Surgeon. 66(12):1179-80, 2000 Dec.
[Case Reports. Journal Article]

UI: 11149595

Authors Full Name

Danikas, D. Theodorou, S J. Matthews, W E. Rienzo, A A.

embase
BIOMEDICAL ANSWERS

Embase Session Results

No.	Query	Results
#27	**#21** AND **#26**	1,116
#26	**#22** OR **#23** OR **#24** OR **#25**	1,089,046
#25	**surviv***:ab,ti	766,227
#24	(**quality** NEAR/3 **life**):ab,ti	167,889
#23	**'quality of life'**/exp	205,230
#22	**'survival'**/exp	456,216
#21	**#1** AND **#20**	2,352
#20	**#8** OR **#19**	203,512
#19	**#9** OR **#10** OR **#11** OR **#12** OR **#13** OR **#14** OR **#17** OR **#18**	91,208
#18	**'radiofrequency ablation'**/exp OR **'catheter ablation'**/exp OR **'chemoembolization'**/exp OR **'cryosurgery'**/exp	41,953
#17	**#15** AND **#16**	275
#16	((**radionuclide** OR **radiation**) NEAR/3 (**treatment** OR **therapy**)):ab,ti	69,899
#15	(**selective** NEAR/3 (**radionuclide** OR **radiation**)):ab,ti	464
#14	**pprt**:ab,ti	20
#13	**biotherapy**:ab,ti OR **emboli?ation**:ab,ti OR **chemoemboli?ation**:ab,ti OR **radioemboli?ation**:ab,ti	48,284
#12	((**radiofrequency** OR **'radio frequency'** OR **rf**) NEAR/3 **ablat***):ab,ti	14,891
#11	**cryosurger***:ab,ti OR **cryoablat***:ab,ti OR **radioablat***:ab,ti OR **thermoablat***:ab,ti	6,404
#10	(**ablative** NEAR/3 **therap***):ab,ti	1,751
#9	(**ethanol** NEAR/3 **injection**):ab,ti	3,027
#8	**#2** OR **#3** OR **#4** OR **#5** OR **#6** OR **#7**	114,575
#7	**'lung cancer'**/exp/dm_su	17,891
#6	**'pancreas cancer'**/exp/dm_su	6,823
#5	**'intestine cancer'**/exp/dm_su	17,834

**#4**	**'intestine'**/exp OR **'pancreas'**/exp OR **'lung'**/exp AND **'cancer surgery'**/exp	**4,283**
**#3**	**'pancreas resection'**/exp OR **'pancreaticoduodenectomy'**/exp OR **'lung resection'**/exp OR **'colon resection'**/exp	**65,196**
**#2**	**pancreatectomy**:de,ab,ti OR **pancreaticoduodenectomy**:de,ab,ti OR **pulmonectomy**:de,ab,ti OR **lobectomy**:de,ab,ti OR **colectomy**:de,ab,ti OR **ileocolectomy**:de,ab,ti OR **pneumonectomy**:de,ab,ti	**49,619**
**#1**		

**#1.43**	**#1.39** NOT **#1.42**	**7,295**
**#1.42**	**#1.40** NOT **#1.41**	
**#1.41**	**#1.35** NOT **#1.38** AND ([newborn]/lim OR [infant]/lim OR [preschool]/lim OR [school]/lim OR [child]/lim OR [adolescent]/lim) AND ([adult]/lim OR [aged]/lim)	
**#1.40**	**#1.35** NOT **#1.38** AND ([newborn]/lim OR [infant]/lim OR [preschool]/lim OR [school]/lim OR [child]/lim OR [adolescent]/lim)	
**#1.39**	**#1.35** NOT **#1.38**	
**#1.38**	**#1.36** NOT **#1.37**	
**#1.37**	**#1.28** AND **#1.34** AND [animals]/lim AND [humans]/lim	
**#1.36**	**#1.28** AND **#1.34** AND [animals]/lim	
**#1.35**	**#1.28** AND **#1.34**	
**#1.34**	**#1.29** OR **#1.32** OR **#1.33**	
**#1.33**	(**metasta*** NEAR/3 (**liver** OR **hepatic**)):ab,ti	
**#1.32**	**#1.30** AND **#1.31**	
**#1.31**	((**tumor*** OR **tumour*** OR **cancer** OR **neoplasm*** OR **adenoma*** OR **carcinoma***) NEAR/10 (**liver** OR **hepatic**)):ab,ti	
**#1.30**	((**secondar*** OR **spread** OR **advanced**) NEAR/3 (**tumor*** OR **tumour*** OR **cancer** OR **neoplasm*** OR **adenoma*** OR **carcinoma***)):ab,ti	
**#1.29**	**'liver metastasis'**/exp	
**#1.28**	**#1.13** OR **#1.27**	
**#1.27**	**#1.14** OR **#1.15** OR **#1.16** OR **#1.19** OR **#1.20** OR **#1.21** OR **#1.24** OR **#1.25** OR **#1.26**	
**#1.26**	((**'verner morrison'** OR **'zollinger ellison'**) NEAR/3 **syndrome**):ab,ti	
**#1.25**	**'pancreatic cholera'**:ab,ti	
**#1.24**	**#1.22** AND **#1.23**	
**#1.23**	(**diarr*** NEAR/3 **syndrome**):ab,ti	
**#1.22**	(**watery** NEAR/3 **diarr***):ab,ti	
**#1.21**	((**diarrheogenic** OR **'vip secreting'** OR **'vip secreting'**) NEAR/3 (**tumor*** OR **tumour***)):ab,ti	
**#1.20**	**familial**:ab,ti AND (**endocrine** NEAR/3 **adenomatos?s**):ab,ti	

**#1.19**	**#1.17** AND **#1.18**
**#1.18**	((**islet** OR **island** OR **beta**) NEAR/3 **cell**):ab,ti
**#1.17**	(**cell** NEAR/2 (**tumor*** OR **tumour*** OR **adenoma*** OR **carcinoma***)):ab,ti
**#1.16**	**multiple**:ab,ti AND (**endocrine** NEAR/3 (**neoplasia*** OR **adenopath*** OR **adenomatos?s** OR **neoplasm***)):ab,ti
**#1.15**	**carcinoid***:ab,ti OR **insulinoma***:ab,ti OR **gastrinoma***:ab,ti OR **glucagonoma***:ab,ti OR **vipoma***:ab,ti OR **somatostatinoma***:ab,ti OR **apudoma***:ab,ti OR **adenoma***:ab,ti OR **nesidioblastoma***:ab,ti OR **argentaffinoma***:ab,ti
**#1.14**	((**neuroendocrine** OR **gastroenteropancreatic**) NEAR/3 (**tumor*** OR **tumour*** OR **neoplasm** OR **adenoma*** OR **carcinoma***)):ab,ti
**#1.13**	**#1.1** OR **#1.2** OR **#1.3** OR **#1.4** OR **#1.5** OR **#1.6** OR **#1.7** OR **#1.8** OR **#1.9** OR **#1.10** OR **#1.11** OR **#1.12**
**#1.12**	**'pancreas tumor'**/exp
**#1.11**	**'multiple endocrine neoplasia'**/exp
**#1.10**	**'vipoma'**/exp
**#1.9**	**'somatostatinoma'**/exp
**#1.8**	**'glucagonoma'**/exp
**#1.7**	**'gastrinoma'**/exp
**#1.6**	**'pancreas islet cell carcinoma'**/exp
**#1.5**	**'insulinoma'**/exp
**#1.4**	**'pancreas islet cell tumor'**/exp
**#1.3**	**'carcinoid'**/exp
**#1.2**	**'apudoma'**/exp
**#1.1**	**'neuroendocrine tumor'**/exp

ELSEVIER Copyright © 2012 Elsevier B.V. All rights reserved. Embase is a trademark of Elsevier B.V.

Search Name: NET-Session_9

Comments: Breitenstein_USZ

Save Date: 2012-05-27 09:00:20.863

ID	Search
#1	MeSH descriptor Neuroendocrine Tumors explode all trees
#2	MeSH descriptor Apudoma explode all trees
#3	MeSH descriptor Carcinoid Tumor explode all trees
#4	MeSH descriptor Adenoma, Islet Cell explode all trees
#5	MeSH descriptor Insulinoma explode all trees
#6	MeSH descriptor Carcinoma, Islet Cell explode all trees
#7	MeSH descriptor Gastrinoma explode all trees
#8	MeSH descriptor Glucagonoma explode all trees
#9	MeSH descriptor Somatostatinoma explode all trees
#10	MeSH descriptor Vipoma explode all trees
#11	MeSH descriptor Multiple Endocrine Neoplasia explode all trees
#12	MeSH descriptor Pancreatic Neoplasms explode all trees
#13	(#1 OR #2 OR #3 OR #4 OR #5 OR #6 OR #7 OR #8 OR #9 OR (#10 AND #11) OR #12)
#14	((neuroendocrine OR gastroenteropancreatic) NEAR/3 (tumor* OR tumour* OR neoplasm OR adenoma* OR carcinoma*)):ti,ab,kw or (carcinoid* or insulinoma* or gastrinoma* or glucagonoma* or vipoma* or somatostatinoma* or apudoma* or adenoma* or nesidioblastoma* or argentaffinoma*):ti,ab,kw or ((multiple endocrine) NEAR/3 (neoplasia* OR adenopath* OR adenomatos?s OR neoplasm*)):ti,ab,kw
#15	((islet or island or beta) NEAR/3 (cell NEAR/2 (tumo?r* or adenoma* or carcinoma*))):ti,ab,kw
#16	((familial endocrine) NEAR/3 adenomatos?s):ti,ab,kw
#17	((diarrheogenic OR 'vip secreting' OR 'vip secreting') NEAR/3 (tumor* OR tumour*)):ti,ab,kw
#18	(watery diarrh$ syndrome):ti,ab,kw
#19	"pancreatic cholera":ti,ab,kw
#20	(('verner morrison' OR 'zollinger ellison') NEAR/3 syndrome):ti,ab,kw
#21	(#14 OR #15)
#22	(#13 OR #21)
#23	MeSH descriptor Neoplasm Metastasis explode all trees
#24	MeSH descriptor Liver explode all trees
#25	(liver or hepatic):ti or (liver or hepatic):ab
#26	(#24 OR #25)
#27	(#23 AND #26)
#28	((secondar* OR spread OR advanced) NEAR/3 (tumor* OR tumour* OR cancer OR neoplasm* OR adenoma* carcinoma*)):ti,ab,kw

#29 ((tumor* OR tumour* OR cancer OR neoplasm* OR adenoma* OR carcinoma*) NEAR/10 (liver OR hepatic)):ti,ab,kw

#30 (#28 AND #29)

#31 (metasta* NEAR/3 (liver OR hepatic)):ti,ab,kw

#32 MeSH descriptor Liver Neoplasms explode all trees with qualifier: SC

#33 (#27 OR #30 OR #31 OR #32)

#34 (#22 AND #33)

#35 MeSH descriptor Melanoma explode all trees

#36 MeSH descriptor Neurilemma explode all trees

#37 (#36 OR #35)

#38 (#34 AND NOT #37)

#39 (pancreatectomy or pancreaticoduodenectomy or pulmonectomy or lobectomy or colectomy or ileocolectomy or pneumonectomy)(pancreatectomy or pancreaticoduodenectomy or pulmonectomy or lobectomy or colectomy or ileocolectomy or pneumonectomy):ti,ab,kw

#40 MeSH descriptor Pancreatectomy explode all trees

#41 MeSH descriptor Pancreaticoduodenectomy explode all trees

#42 MeSH descriptor Pneumonectomy explode all trees

#43 MeSH descriptor Colectomy explode all trees

#44 MeSH descriptor Ileum explode all trees with qualifier: SU

#45 MeSH descriptor Pancreas explode all trees with qualifier: SU

#46 MeSH descriptor Lung explode all trees with qualifier: SU

#47 MeSH descriptor Intestines explode all trees with qualifier: SU

#48 MeSH descriptor Pancreatic Neoplasms explode all trees with qualifier: SU

#49 MeSH descriptor Lung Neoplasms explode all trees with qualifier: SU

#50 MeSH descriptor Intestinal Neoplasms explode all trees with qualifier: SU

#51 (#39 OR #40 OR #41 OR #42 OR #43 OR #44 OR #45 OR #46 OR #47 OR #48 OR #49 OR #50)

#52 (ethanol NEAR/3 injection):ti,ab,kw or (ablative NEAR/3 therap*):ti,ab,kw

#53 (crosurger* or cryoablat* or radioablat* or thermoablat*):ti,ab,kw or ((radiofrequency OR 'radio frequency' OR rf) NEAR/3 ablat*):ti,ab,kw or (biotherapy or emboli?ation or chemoemboli?ation or radioemboli?ation):ti,ab,kw or (pprt):ti,ab,kw

#54 (selective NEAR/3 (radionuclide OR radiation)):ti,ab,kw and ((radionuclide OR radiation) NEAR/3 (treatment OR therapy)):ti,ab,kw

#55 MeSH descriptor Catheter Ablation explode all trees

#56 MeSH descriptor Ablation Techniques explode all trees

#57 MeSH descriptor Chemoembolization, Therapeutic explode all trees

#58 MeSH descriptor Cryosurgery explode all trees

#59 (#52 OR #53 OR #54 OR #55 OR #56 OR #57 OR #58)

#60 (#51 OR #59)

#61 (#38 AND #60)

#62 MeSH descriptor Survival Rate explode all trees

#63 MeSH descriptor Survival Analysis explode all trees

#64 MeSH descriptor Disease-Free Survival explode all trees

#65 MeSH descriptor Quality of Life explode all trees

#66 (quality NEAR/3 life):ti,ab,kw or (surviv*):ti,ab,kw

#67 (#62 OR #63 OR #64 OR #65 OR #66)

#68 (#61 AND #67)

ID	Search	Hits	Edit	Delete
#1	MeSH descriptor **Neuroendocrine Tumors** explode all trees	1156	edit	delete
#2	MeSH descriptor **Apudoma** explode all trees	0	edit	delete
#3	MeSH descriptor **Carcinoid Tumor** explode all trees	44	edit	delete
#4	MeSH descriptor **Adenoma, Islet Cell** explode all trees	15	edit	delete
#5	MeSH descriptor **Insulinoma** explode all trees	9	edit	delete
#6	MeSH descriptor **Carcinoma, Islet Cell** explode all trees	6	edit	delete
#7	MeSH descriptor **Gastrinoma** explode all trees	3	edit	delete
#8	MeSH descriptor **Glucagonoma** explode all trees	0	edit	delete
#9	MeSH descriptor **Somatostatinoma** explode all trees	1	edit	delete
#10	MeSH descriptor **Vipoma** explode all trees	2	edit	delete
#11	MeSH descriptor **Multiple Endocrine Neoplasia** explode all trees	7	edit	delete
#12	MeSH descriptor **Pancreatic Neoplasms** explode all trees	690	edit	delete
#13	(#1 OR #2 OR #3 OR #4 OR #5 OR #6 OR #7 OR #8 OR #9 OR (#10 AND #11) OR #12)	1822	edit	delete
#14	((neuroendocrine OR gastroenteropancreatic) NEAR/3 (tumor* OR tumour* OR neoplasm OR adenoma* OR carcinoma*)):ti,ab,kw or (carcinoid* or insulinoma* or gastrinoma* or glucagonoma* or vipoma* or somatostatinoma* or apudoma* or adenoma* or nesidioblastoma* or argentaffinoma*):ti,ab,kw or ((multiple endocrine) NEAR/3 (neoplasia* OR adenopath* OR adenomatos?s OR neoplasm*)):ti,ab,kw	1304	edit	delete
#15	((islet or island or beta) NEAR/3 (cell NEAR/2 (tumo?r* or adenoma* or carcinoma*))):ti,ab,kw	15	edit	delete
#16	((familial endocrine) NEAR/3 adenomatos?s):ti,ab,kw	0	edit	delete
#17	((diarrheogenic OR 'vip secreting' OR 'vip secreting') NEAR/3 (tumor* OR tumour*)):ti,ab,kw	0	edit	delete
#18	(watery diarrh$ syndrome):ti,ab,kw	0	edit	delete
#19	"pancreatic cholera":ti,ab,kw	0	edit	delete
#20	(('verner morrison' OR 'zollinger ellison') NEAR/3 syndrome):ti,ab,kw	0	edit	delete
#21	(#14 OR #15)	1310	edit	delete
#22	(#13 OR #21)	3010	edit	delete
#23	MeSH descriptor **Neoplasm Metastasis** explode all trees	3275	edit	delete
#24	MeSH descriptor **Liver** explode all trees	2339	edit	delete
#25	(liver or hepatic):ti or (liver or hepatic):ab	24742	edit	delete
#26	(#24 OR #25)	25211	edit	delete
#27	(#23 AND #26)	185	edit	delete
#28	((secondar* OR spread OR advanced) NEAR/3 (tumor* OR tumour* OR cancer OR neoplasm* OR adenoma* carcinoma*)):ti,ab,kw	7713	edit	delete
#29	((tumor* OR tumour* OR cancer OR neoplasm* OR adenoma* OR carcinoma*) NEAR/10 (liver OR hepatic)):ti,ab,kw	2779	edit	delete
#30	(#28 AND #29)	612	edit	delete
#31	(metasta* NEAR/3 (liver OR hepatic)):ti,ab,kw	791	edit	delete
#32	MeSH descriptor **Liver Neoplasms** explode all trees with qualifier: **SC**	530	edit	delete
#33	(#27 OR #30 OR #31 OR #32)	1219	edit	delete
#34	(#22 AND #33)	101	edit	delete
#35	MeSH descriptor **Melanoma** explode all trees	952	edit	delete
#36	MeSH descriptor **Neurilemma** explode all trees	1	edit	delete

#37	(#36 OR #35)	954	edit	delete
#38	(#34 AND NOT #37)	69	edit	delete
#39	(pancreatectomy or pancreaticoduodenectomy or pulmonectomy or lobectomy or colectomy or ileocolectomy or pneumonectomy)(pancreatectomy or pancreaticoduodenectomy or pulmonectomy or lobectomy or colectomy or ileocolectomy or pneumonectomy):ti,ab,kw	1433	edit	delete
#40	MeSH descriptor **Pancreatectomy** explode all trees	109	edit	delete
#41	MeSH descriptor **Pancreaticoduodenectomy** explode all trees	150	edit	delete
#42	MeSH descriptor **Pneumonectomy** explode all trees	386	edit	delete
#43	MeSH descriptor **Colectomy** explode all trees	560	edit	delete
#44	MeSH descriptor **Ileum** explode all trees with qualifier: **SU**	116	edit	delete
#45	MeSH descriptor **Pancreas** explode all trees with qualifier: **SU**	86	edit	delete
#46	MeSH descriptor **Lung** explode all trees with qualifier: **SU**	104	edit	delete
#47	MeSH descriptor **Intestines** explode all trees with qualifier: **SU**	1158	edit	delete
#48	MeSH descriptor **Pancreatic Neoplasms** explode all trees with qualifier: **SU**	212	edit	delete
#49	MeSH descriptor **Lung Neoplasms** explode all trees with qualifier: **SU**	534	edit	delete
#50	MeSH descriptor **Intestinal Neoplasms** explode all trees with qualifier: **SU**	1448	edit	delete
#51	(**#39** OR **#40** OR **#41** OR **#42** OR **#43** OR **#44** OR **#45** OR **#46** OR **#47** OR **#48** OR **#49** OR **#50**)	4172	edit	delete
#52	(ethanol NEAR/3 injection):ti,ab,kw or (ablative NEAR/3 therap*):ti,ab,kw	176	edit	delete
#53	(crosurger* or cryoablat* or radioablat* or thermoablat*):ti,ab,kw or ((radiofrequency OR 'radio frequency' OR rf) NEAR/3 ablat*):ti,ab,kw or (biotherapy or emboli?ation or chemoemboli?ation or radioemboli?ation):ti,ab,kw or (pprt):ti,ab,kw	1504	edit	delete
#54	(selective NEAR/3 (radionuclide OR radiation)):ti,ab,kw and ((radionuclide OR radiation) NEAR/3 (treatment OR therapy)):ti,ab,kw	10	edit	delete
#55	MeSH descriptor **Catheter Ablation** explode all trees	915	edit	delete
#56	MeSH descriptor **Ablation Techniques** explode all trees	4101	edit	delete
#57	MeSH descriptor **Chemoembolization, Therapeutic** explode all trees	192	edit	delete
#58	MeSH descriptor **Cryosurgery** explode all trees	253	edit	delete
#59	(#52 OR #53 OR #54 OR #55 OR #56 OR #57 OR #58)	5267	edit	delete
#60	(#51 OR #59)	9384	edit	delete
#61	(#38 AND #60)	21	edit	delete
#62	MeSH descriptor **Survival Rate** explode all trees	7198	edit	delete
#63	MeSH descriptor **Survival Analysis** explode all trees	12895	edit	delete
#64	MeSH descriptor **Disease-Free Survival** explode all trees	3635	edit	delete
#65	MeSH descriptor **Quality of Life** explode all trees	11889	edit	delete
#66	(quality NEAR/3 life):ti,ab,kw or (surviv*):ti,ab,kw	56737	edit	delete
#67	(#62 OR #63 OR #64 OR #65 OR #66)	58558	edit	delete
#68	(#61 AND #67)	16	edit	delete

Results of Literature Search

NET and Liver Metastases – Session 10

Search Protocols:
Session_10_Ovid_ Search Results.pdf
Session_10_EMBASE.pdf
Session_10_Cochrane.docx

	Time span	References	References after Deduplication
**Medline/Premedline**	no limit	612	600
**Embase**	no limit	1004	528
**Cochrane**	no limit	11	1
**Pool**		1627	1129

References in _Session_10.enlx_

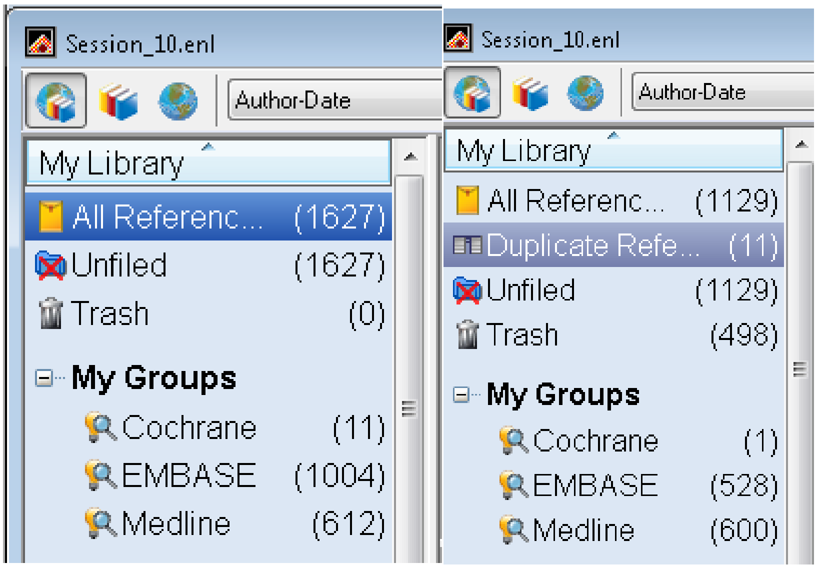

Database(s): **Ovid MEDLINE(R), Ovid MEDLINE(R) In-Process & Other Non-Indexed Citations, Ovid MEDLINE(R) Daily and Ovid OLDMEDLINE(R)** 1946 to Present

Search Strategy:

#	Searches	Results
1	exp Neuroendocrine Tumors/ not (exp melanoma/ or exp neurilemmoma/)	39296
2	exp Apudoma/	443
3	exp Carcinoid Tumor/	10844
4	exp Adenoma, Islet Cell/	7319
5	exp Insulinoma/	3679
6	exp Carcinoma, Islet Cell/	2146
7	exp Gastrinoma/	838
8	exp Glucagonoma/	665
9	exp Somatostatinoma/	283
10	exp Vipoma/	412
11	exp Multiple Endocrine Neoplasia/	4325
12	exp Pancreatic Neoplasms/	49120
13	or/1-12	87853
14	((neuroendocrine or gastroenteropancreatic) adj3 (tumo?r$ or neoplasm or adenoma$ or carcinoma$)).ti,ab.	8089
15	(carcinoid$ or insulinoma$ or gastrinoma$ or glucagonoma$ or vipoma$ or somatostatinoma$ or apudoma$ or adenoma$ or nesidioblastoma$ or argentaffinoma$).ti,ab.	78242
16	(multiple endocrine adj3 (neoplasia$ or adenopath$ or adenomatos?s or neoplasm$)).ti,ab.	4319
17	((islet or island or beta) adj3 (cell adj2 (tumo?r$ or adenoma$ or carcinoma$))).ti,ab.	2655
18	(familial endocrine adj3 adenomatos?s).ti,ab.	8
19	((diarrheogenic or VIP secreting) adj3 tumo?r$).ti,ab.	39
20	watery diarrh$ syndrome.ti,ab.	58
21	"pancreatic cholera".ti,ab.	59
22	((verner morrison or zollinger ellison) adj3 syndrome).ti,ab.	2085
23	or/14-22	89360
24	13 or 23	152055
25	exp Liver Neoplasms/sc [Secondary]	23515
26	exp Neoplasm Metastasis/	141543
27	exp Liver/	366332

28	(liver or hepatic).ti,ab.	649227
29	27 or 28	759333
30	26 and 29	10559
31	((secondar$ or spread or advanced) adj3 (tumo?r$ or cancer or neoplasm$ or adenoma$ or carcinoma$)).ti,ab.	66963
32	((tumo?r$ or cancer or neoplasm$ or adenoma$ or carcinoma$) adj10 (liver or hepatic)).ti,ab.	68275
33	31 and 32	3529
34	((liver or hepatic) adj3 metasta*).ti,ab.	23489
35	25 or 30 or 33 or 34	40318
36	24 and 35	6008
37	limit 36 to animals	514
38	limit 37 to humans	318
39	37 not 38	196
40	36 not 39	5812
41	limit 40 to "all child (0 to 18 years)"	426
42	limit 41 to "all adult (19 plus years)"	349
43	41 not 42	77
44	40 not 43	5735
45	exp Hepatectomy/	19362
46	exp Liver Neoplasms/sc and exp Liver Neoplasms/su [Secondary,Surgery]	5189
47	((resection or segmentectomy or metastasectomy or surgery) adj5 (liver or hepatic)).ti,ab.	15934
48	45 or 46 or 47	30292
49	ethanol injection.ti,ab.	1729
50	(ablative adj3 therap*).ti,ab.	1307
51	(cryosurg* or cryoablat* or radioablat* or thermoablat* or microwave).ti,ab.	22209
52	((radiofrequency or radio-frequency or RF) adj3 ablat*).ti,ab.	10721
53	(biotherapy or emboli?ation or chemoemboli?ation or radioemboli?ation or chemo-emboli?ation or radio-emboli?ation).ti,ab.	32355
54	pprt.ti,ab.	11
55	(selective adj3 (radionuclide or radiation)).ti,ab.	357
56	((radionuclide or radiation) adj3 (treatment or therapy)).ti,ab.	55050
57	55 and 56	194
58	exp Catheter Ablation/ or exp Ablation Techniques/	80694
59	exp Cryosurgery/	10363
60	exp Chemoembolization, Therapeutic/	2897

61	49 or 50 or 51 or 52 or 53 or 54 or 57 or 58 or 60	133558
62	48 or 61	160170
63	44 and 62	1308
64	exp Disease-Free Survival/ or exp Survival Analysis/ or exp Survival/ or exp Survival Rate/	247642
65	exp "Quality of Life"/	99085
66	(quality adj3 life).ti,ab.	121923
67	surviv*.mp. [mp=title, abstract, original title, name of substance word, subject heading word, protocol supplementary concept, rare disease supplementary concept, unique identifier]	798042
68	or/64-67	950290
69	63 and 68	602
70	exp Liver Transplantation/	39273
71	((liver or hepatic) adj5 (transplant* or graft* or donat* or donor*)).ti,ab.	44472
72	70 or 71	52087
73	62 or 72	207043
74	44 and 73	1353
75	68 and 74	612

1. **Treatment of liver metastases in patients with neuroendocrine tumors of gastroesophageal and pancreatic origin.**

   Gu P. Wu J. Newman E. Muggia F.

   International Journal of Hepatology. 2012:131659, 2012.
   [Journal Article]

   UI: 22518318

   Authors Full Name

   Gu, Ping. Wu, Jennifer. Newman, Elliot. Muggia, Franco.

Embase Session Results

No.	Query	Results
#30	**#23** AND **#29**	1,004
#29	**#1** AND **#28**	2,036
#28	**#17** OR **#27**	210,870
#27	**#25** OR **#26**	71,604
#26	((**liver** OR **hepatic**) NEAR/5 (**transplant*** OR **graft*** OR **donat*** OR **donor***)):ab,ti	58,105
#25	**'liver transplantation'**/exp	61,081
#24	**#18** AND **#23**	980
#23	**#19** OR **#20** OR **#21** OR **#22**	1,086,668
#22	**surviv***:ab,ti	764,334
#21	(**quality** NEAR/3 **life**):ab,ti	167,597
#20	**'quality of life'**/exp	204,915
#19	**'survival'**/exp	454,858
#18	**#1** AND **#17**	1,962
#17	**#5** OR **#16**	146,936
#16	**#6** OR **#7** OR **#8** OR **#9** OR **#10** OR **#11** OR **#14** OR **#15**	111,176
#15	**'radiofrequency ablation'**/exp OR **'catheter ablation'**/exp OR **'chemoembolization'**/exp OR **'cryosurgery'**/exp	41,866
#14	**#12** AND **#13**	273
#13	((**radionuclide** OR **radiation**) NEAR/3 (**treatment** OR **therapy**)):ab,ti	69,818
#12	(**selective** NEAR/3 (**radionuclide** OR **radiation**)):ab,ti	462
#11	**pprt**:ab,ti	20
#10	**biotherapy**:ab,ti OR **emboli?ation**:ab,ti OR **chemoemboli?ation**:ab,ti OR **radioemboli?ation**:ab,ti OR **'chemo emboli?ation'**:ab,ti OR **'radio emboli?ation'**:ab,ti	48,194
#9	((**radiofrequency** OR **'radio frequency'** OR **rf**) NEAR/3 **ablat***):ab,ti	14,858
#8	**cryosurg***:ab,ti OR **cryoablat***:ab,ti OR **radioablat***:ab,ti OR **thermoablat***:ab,ti OR	27,553

	**microwave**:ab,ti	
#7	(**ablative** NEAR/3 **therap***):ab,ti	**1,748**
#6	(**ethanol** NEAR/3 **injection**):ab,ti	**3,025**
#5	**#2** OR **#3** OR **#4**	**40,834**
#4	((**resection** OR **segmentectomy** OR **metastasectomy** OR **surgery**) NEAR/5 (**liver** OR **hepatic**)):ab,ti	**20,380**
#3	**'liver metastasis'**/exp/dm_su	**5,080**
#2	**'liver resection'**/exp	**30,026**
#1		

**#1.43**	**#1.39** NOT **#1.42**	**7,288**
**#1.42**	**#1.40** NOT **#1.41**	
**#1.41**	**#1.35** NOT **#1.38** AND ([newborn]/lim OR [infant]/lim OR [preschool]/lim OR [school]/lim OR [child]/lim OR [adolescent]/lim) AND ([adult]/lim OR [aged]/lim)	
**#1.40**	**#1.35** NOT **#1.38** AND ([newborn]/lim OR [infant]/lim OR [preschool]/lim OR [school]/lim OR [child]/lim OR [adolescent]/lim)	
**#1.39**	**#1.35** NOT **#1.38**	
**#1.38**	**#1.36** NOT **#1.37**	
**#1.37**	**#1.28** AND **#1.34** AND [animals]/lim AND [humans]/lim	
**#1.36**	**#1.28** AND **#1.34** AND [animals]/lim	
**#1.35**	**#1.28** AND **#1.34**	
**#1.34**	**#1.29** OR **#1.32** OR **#1.33**	
**#1.33**	(**metasta*** NEAR/3 (**liver** OR **hepatic**)):ab,ti	
**#1.32**	**#1.30** AND **#1.31**	
**#1.31**	((**tumor*** OR **tumour*** OR **cancer** OR **neoplasm*** OR **adenoma*** OR **carcinoma***) NEAR/10 (**liver** OR **hepatic**)):ab,ti	
**#1.30**	((**secondar*** OR **spread** OR **advanced**) NEAR/3 (**tumor*** OR **tumour*** OR **cancer** OR **neoplasm*** OR **adenoma*** OR **carcinoma***)):ab,ti	
**#1.29**	**'liver metastasis'**/exp	
**#1.28**	**#1.13** OR **#1.27**	
**#1.27**	**#1.14** OR **#1.15** OR **#1.16** OR **#1.19** OR **#1.20** OR **#1.21** OR **#1.24** OR **#1.25** OR **#1.26**	
**#1.26**	((**'verner morrison'** OR **'zollinger ellison'**) NEAR/3 **syndrome**):ab,ti	
**#1.25**	**'pancreatic cholera'**:ab,ti	
**#1.24**	**#1.22** AND **#1.23**	
**#1.23**	(**diarr*** NEAR/3 **syndrome**):ab,ti	
**#1.22**	(**watery** NEAR/3 **diarr***):ab,ti	

**#1.21**	((**diarrheogenic** OR **'vip secreting'** OR **'vip secreting'**) NEAR/3 (**tumor*** OR **tumour***)):ab,ti
**#1.20**	**familial**:ab,ti AND (**endocrine** NEAR/3 **adenomatos?s**):ab,ti
**#1.19**	**#1.17** AND **#1.18**
**#1.18**	((**islet** OR **island** OR **beta**) NEAR/3 **cell**):ab,ti
**#1.17**	(**cell** NEAR/2 (**tumor*** OR **tumour*** OR **adenoma*** OR **carcinoma***)):ab,ti
**#1.16**	**multiple**:ab,ti AND (**endocrine** NEAR/3 (**neoplasia*** OR **adenopath*** OR **adenomatos?s** OR **neoplasm***)):ab,ti
**#1.15**	**carcinoid***:ab,ti OR **insulinoma***:ab,ti OR **gastrinoma***:ab,ti OR **glucagonoma***:ab,ti OR **vipoma***:ab,ti OR **somatostatinoma***:ab,ti OR **apudoma***:ab,ti OR **adenoma***:ab,ti OR **nesidioblastoma***:ab,ti OR **argentaffinoma***:ab,ti
**#1.14**	((**neuroendocrine** OR **gastroenteropancreatic**) NEAR/3 (**tumor*** OR **tumour*** OR **neoplasm** OR **adenoma*** OR **carcinoma***)):ab,ti
**#1.13**	**#1.1** OR **#1.2** OR **#1.3** OR **#1.4** OR **#1.5** OR **#1.6** OR **#1.7** OR **#1.8** OR **#1.9** OR **#1.10** OR **#1.11** OR **#1.12**
**#1.12**	**'pancreas tumor'**/exp
**#1.11**	**'multiple endocrine neoplasia'**/exp
**#1.10**	**'vipoma'**/exp
**#1.9**	**'somatostatinoma'**/exp
**#1.8**	**'glucagonoma'**/exp
**#1.7**	**'gastrinoma'**/exp
**#1.6**	**'pancreas islet cell carcinoma'**/exp
**#1.5**	**'insulinoma'**/exp
**#1.4**	**'pancreas islet cell tumor'**/exp
**#1.3**	**'carcinoid'**/exp
**#1.2**	**'apudoma'**/exp
**#1.1**	**'neuroendocrine tumor'**/exp

ELSEVIER Copyright © 2012 Elsevier B.V. All rights reserved. Embase is a trademark of Elsevier B.V.

Search Name: Net Session_10

Comments: Breitenstein USZ

Save Date: 2012-05-23 10:27:26.173

ID	Search
#1	MeSH descriptor Neuroendocrine Tumors explode all trees
#2	MeSH descriptor Apudoma explode all trees
#3	MeSH descriptor Carcinoid Tumor explode all trees
#4	MeSH descriptor Adenoma, Islet Cell explode all trees
#5	MeSH descriptor Insulinoma explode all trees
#6	MeSH descriptor Carcinoma, Islet Cell explode all trees
#7	MeSH descriptor Gastrinoma explode all trees
#8	MeSH descriptor Glucagonoma explode all trees
#9	MeSH descriptor Somatostatinoma explode all trees
#10	MeSH descriptor Vipoma explode all trees
#11	MeSH descriptor Multiple Endocrine Neoplasia explode all trees
#12	MeSH descriptor Pancreatic Neoplasms explode all trees
#13	(#1 OR #2 OR #3 OR #4 OR #5 OR #6 OR #7 OR #8 OR #9 OR (#10 AND #11) OR #12)
#14	((neuroendocrine OR gastroenteropancreatic) NEAR/3 (tumor* OR tumour* OR neoplasm OR adenoma* OR carcinoma*)):ti,ab,kw or (carcinoid* or insulinoma* or gastrinoma* or glucagonoma* or vipoma* or somatostatinoma* or apudoma* or adenoma* or nesidioblastoma* or argentaffinoma*):ti,ab,kw or ((multiple endocrine) NEAR/3 (neoplasia* OR adenopath* OR adenomatos?s OR neoplasm*)):ti,ab,kw
#15	((islet or island or beta) NEAR/3 (cell NEAR/2 (tumo?r* or adenoma* or carcinoma*))):ti,ab,kw
#16	((familial endocrine) NEAR/3 adenomatos?s):ti,ab,kw
#17	((diarrheogenic OR 'vip secreting' OR 'vip secreting') NEAR/3 (tumor* OR tumour*)):ti,ab,kw
#18	(watery diarrh$ syndrome):ti,ab,kw
#19	"pancreatic cholera":ti,ab,kw
#20	(('verner morrison' OR 'zollinger ellison') NEAR/3 syndrome):ti,ab,kw
#21	(#14 OR #15)
#22	(#13 OR #21)
#23	MeSH descriptor Neoplasm Metastasis explode all trees
#24	MeSH descriptor Liver explode all trees
#25	(liver or hepatic):ti or (liver or hepatic):ab
#26	(#24 OR #25)
#27	(#23 AND #26)
#28	((secondar* OR spread OR advanced) NEAR/3 (tumor* OR tumour* OR cancer OR neoplasm* OR adenoma* carcinoma*)):ti,ab,kw

#29 ((tumor* OR tumour* OR cancer OR neoplasm* OR adenoma* OR carcinoma*) NEAR/10 (liver OR hepatic)):ti,ab,kw

#30 (#28 AND #29)

#31 (metasta* NEAR/3 (liver OR hepatic)):ti,ab,kw

#32 MeSH descriptor Liver Neoplasms explode all trees with qualifier: SC

#33 (#27 OR #30 OR #31 OR #32)

#34 (#22 AND #33)

#35 MeSH descriptor Melanoma explode all trees

#36 MeSH descriptor Neurilemma explode all trees

#37 (#36 OR #35)

#38 (#34 AND NOT #37)

#39 MeSH descriptor Hepatectomy explode all trees

#40 MeSH descriptor Liver Neoplasms explode all trees with qualifier: SC

#41 MeSH descriptor Liver Neoplasms explode all trees with qualifier: SU

#42 (#40 AND #41)

#43 MeSH descriptor Cryosurgery explode all trees

#44 ((resection OR segmentectomy OR metastasectomy OR surgery) NEAR/5 (liver OR hepatic)):ti,ab,kw

#45 (#39 OR #42 OR #43 OR #44)

#46 (ethanol NEAR/3 injection):ti,ab,kw or (ablative NEAR/3 therap*):ti,ab,kw

#47 (crosurger* or cryoablat* or radioablat* or thermoablat*):ti,ab,kw or ((radiofrequency OR 'radio frequency' OR rf) NEAR/3 ablat*):ti,ab,kw or (biotherapy or emboli?ation or chemoemboli?ation or radioemboli?ation or chemo-emboli?ation or radio-emboli?ation):ti,ab,kw or (pprt):ti,ab,kw

#48 (selective NEAR/3 (radionuclide OR radiation)):ti,ab,kw and ((radionuclide OR radiation) NEAR/3 (treatment OR therapy)):ti,ab,kw

#49 MeSH descriptor Catheter Ablation explode all trees

#50 MeSH descriptor Ablation Techniques explode all trees

#51 MeSH descriptor Chemoembolization, Therapeutic explode all trees

#52 (#46 OR #47 OR #48 OR #49 OR #50 OR #51)

#53 (#45 OR #52)

#54 (#38 AND #53)

#55 MeSH descriptor Survival Rate explode all trees

#56 MeSH descriptor Survival Analysis explode all trees

#57 MeSH descriptor Disease-Free Survival explode all trees

#58 MeSH descriptor Quality of Life explode all trees

#59 (quality NEAR/3 life):ti,ab,kw or (surviv*):ti,ab,kw

#60 (#55 OR #56 OR #57 OR #58 OR #59)

#61 (#54 AND #60)

#62 ((liver OR hepatic) NEAR/5 (transplant* OR graft* OR donat* OR donor*)):ti,ab,kw

#63 MeSH descriptor Liver Transplantation explode all trees

#64 (#62 OR #63)

#65 (#53 OR #64)

#66 (#38 AND #65)

#67 (#66 AND #60)

ID	Search	Hits	Edit	Delete
#1	MeSH descriptor **Neuroendocrine Tumors** explode all trees	1156	edit	delete
#2	MeSH descriptor **Apudoma** explode all trees	0	edit	delete
#3	MeSH descriptor **Carcinoid Tumor** explode all trees	44	edit	delete
#4	MeSH descriptor **Adenoma, Islet Cell** explode all trees	15	edit	delete
#5	MeSH descriptor **Insulinoma** explode all trees	9	edit	delete
#6	MeSH descriptor **Carcinoma, Islet Cell** explode all trees	6	edit	delete
#7	MeSH descriptor **Gastrinoma** explode all trees	3	edit	delete
#8	MeSH descriptor **Glucagonoma** explode all trees	0	edit	delete
#9	MeSH descriptor **Somatostatinoma** explode all trees	1	edit	delete
#10	MeSH descriptor **Vipoma** explode all trees	2	edit	delete
#11	MeSH descriptor **Multiple Endocrine Neoplasia** explode all trees	7	edit	delete
#12	MeSH descriptor **Pancreatic Neoplasms** explode all trees	690	edit	delete
#13	(#1 OR #2 OR #3 OR #4 OR #5 OR #6 OR #7 OR #8 OR #9 OR (#10 AND #11) OR #12)	1822	edit	delete
#14	((neuroendocrine OR gastroenteropancreatic) NEAR/3 (tumor* OR tumour* OR neoplasm OR adenoma* OR carcinoma*)):ti,ab,kw or (carcinoid* or insulinoma* or gastrinoma* or glucagonoma* or vipoma* or somatostatinoma* or apudoma* or adenoma* or nesidioblastoma* or argentaffinoma*):ti,ab,kw or ((multiple endocrine) NEAR/3 (neoplasia* OR adenopath* OR adenomatos?s OR neoplasm*)):ti,ab,kw	1304	edit	delete
#15	((islet or island or beta) NEAR/3 (cell NEAR/2 (tumo?r* or adenoma* or carcinoma*))):ti,ab,kw	15	edit	delete
#16	((familial endocrine) NEAR/3 adenomatos?s):ti,ab,kw	0	edit	delete
#17	((diarrheogenic OR 'vip secreting' OR 'vip secreting') NEAR/3 (tumor* OR tumour*)):ti,ab,kw	0	edit	delete
#18	(watery diarrh$ syndrome):ti,ab,kw	0	edit	delete
#19	"pancreatic cholera":ti,ab,kw	0	edit	delete
#20	(('verner morrison' OR 'zollinger ellison') NEAR/3 syndrome):ti,ab,kw	0	edit	delete
#21	(#14 OR #15)	1310	edit	delete
#22	(#13 OR #21)	3010	edit	delete
#23	MeSH descriptor **Neoplasm Metastasis** explode all trees	3275	edit	delete
#24	MeSH descriptor **Liver** explode all trees	2339	edit	delete
#25	(liver or hepatic):ti or (liver or hepatic):ab	24742	edit	delete

#26	(#24 OR #25)	25211	edit	delete
#27	(#23 AND #26)	185	edit	delete
#28	((secondar* OR spread OR advanced) NEAR/3 (tumor* OR tumour* OR cancer OR neoplasm* OR adenoma* carcinoma*)):ti,ab,kw	7713	edit	delete
#29	((tumor* OR tumour* OR cancer OR neoplasm* OR adenoma* OR carcinoma*) NEAR/10 (liver OR hepatic)):ti,ab,kw	2779	edit	delete
#30	(#28 AND #29)	612	edit	delete
#31	(metasta* NEAR/3 (liver OR hepatic)):ti,ab,kw	791	edit	delete
#32	MeSH descriptor **Liver Neoplasms** explode all trees with qualifier: **SC**	530	edit	delete
#33	(#27 OR #30 OR #31 OR #32)	1219	edit	delete
#34	(#22 AND #33)	101	edit	delete
#35	MeSH descriptor **Melanoma** explode all trees	952	edit	delete
#36	MeSH descriptor **Neurilemma** explode all trees	1	edit	delete
#37	(#36 OR #35)	954	edit	delete
#38	(#34 AND NOT #37)	69	edit	delete
#39	MeSH descriptor **Hepatectomy** explode all trees	404	edit	delete
#40	MeSH descriptor **Liver Neoplasms** explode all trees with qualifier: **SC**	530	edit	delete
#41	MeSH descriptor **Liver Neoplasms** explode all trees with qualifier: **SU**	410	edit	delete
#42	(**#40** AND **#41**)	99	edit	delete
#43	MeSH descriptor **Cryosurgery** explode all trees	253	edit	delete
#44	((resection OR segmentectomy OR metastasectomy OR surgery) NEAR/5 (liver OR hepatic)):ti,ab,kw	1345	edit	delete
#45	(#39 OR #42 OR #43 OR #44)	1659	edit	delete
#46	(ethanol NEAR/3 injection):ti,ab,kw or (ablative NEAR/3 therap*):ti,ab,kw	176	edit	delete
#47	(crosurger* or cryoablat* or radioablat* or thermoablat*):ti,ab,kw or ((radiofrequency OR 'radio frequency' OR rf) NEAR/3 ablat*):ti,ab,kw or (biotherapy or emboli?ation or chemoemboli?ation or radioemboli?ation or chemo-emboli?ation or radio-emboli?ation):ti,ab,kw or (pprt):ti,ab,kw	1504	edit	delete
#48	(selective NEAR/3 (radionuclide OR radiation)):ti,ab,kw and ((radionuclide OR radiation) NEAR/3 (treatment OR therapy)):ti,ab,kw	10	edit	delete
#49	MeSH descriptor **Catheter Ablation** explode all trees	915	edit	delete
#50	MeSH descriptor **Ablation Techniques** explode all trees	4101	edit	delete
#51	MeSH descriptor **Chemoembolization, Therapeutic** explode all trees	192	edit	delete
#52	(#46 OR #47 OR #48 OR #49 OR #50 OR #51)	5267	edit	delete
#53	(#45 OR #52)	6515	edit	delete
#54	(#38 AND #53)	18	edit	delete
#55	MeSH descriptor **Survival Rate** explode all trees	7198	edit	delete
#56	MeSH descriptor **Survival Analysis** explode all trees	12895	edit	delete
#57	MeSH descriptor **Disease-Free Survival** explode all trees	3635	edit	delete
#58	MeSH descriptor **Quality of Life** explode all trees	11889	edit	delete
#59	(quality NEAR/3 life):ti,ab,kw or (surviv*):ti,ab,kw	56737	edit	delete
#60	(#55 OR #56 OR #57 OR #58 OR #59)	58558	edit	delete
#61	(#54 AND #60)	11	edit	delete

#62	((liver OR hepatic) NEAR/5 (transplant* OR graft* OR donat* OR donor*)):ti,ab,kw	2312	edit	delete
#63	MeSH descriptor **Liver Transplantation** explode all trees	1072	edit	delete
#64	(#62 OR #63)	2312	edit	delete
#65	(#53 OR #64)	8528	edit	delete
#66	(#38 AND #65)	18	edit	delete
#67	(#66 AND #60)	11	edit	delete

Results of Literature Search

NET and Liver Metastases – Session 11

Search Protocols:
Session_11_Ovid_ Search Results.pdf
Session_11_EMBASE.pdf
Session_11_Cochrane.docx

	Time span	References	References after Deduplication
**Medline/Premedline**	no limit	56	56
**Embase**	no limit	95	59
**Cochrane**	no limit	3	0
**Pool**		154	115

References in *Session_11.enlx*

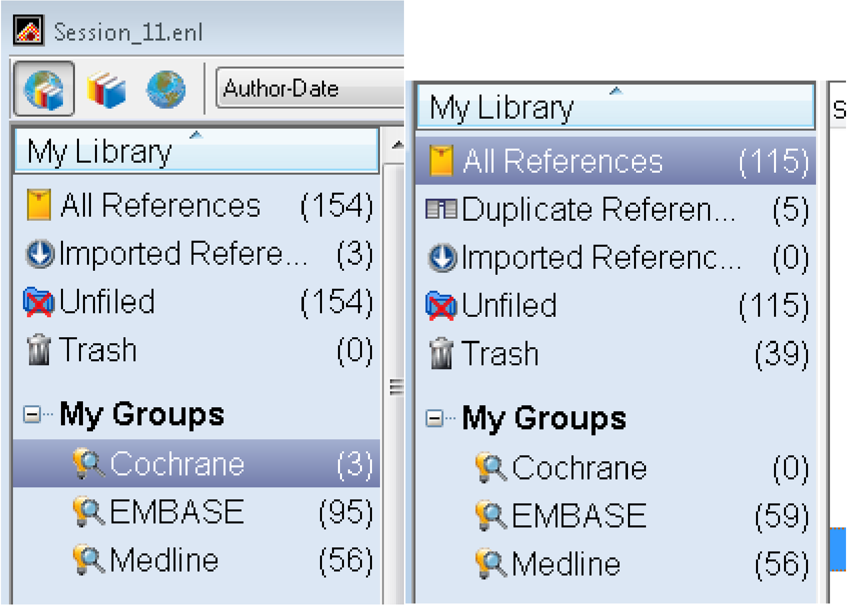

Database(s): **Ovid MEDLINE(R), Ovid MEDLINE(R) In-Process & Other Non-Indexed Citations, Ovid MEDLINE(R) Daily and Ovid OLDMEDLINE(R)** 1946 to Present

Search Strategy:

#	Searches	Results
1	exp Neuroendocrine Tumors/ not (exp melanoma/ or exp neurilemmoma/)	39308
2	exp Apudoma/	443
3	exp Carcinoid Tumor/	10848
4	exp Adenoma, Islet Cell/	7321
5	exp Insulinoma/	3681
6	exp Carcinoma, Islet Cell/	2146
7	exp Gastrinoma/	838
8	exp Glucagonoma/	665
9	exp Somatostatinoma/	283
10	exp Vipoma/	412
11	exp Multiple Endocrine Neoplasia/	4327
12	exp Pancreatic Neoplasms/	49169
13	or/1-12	87915
14	((neuroendocrine or gastroenteropancreatic) adj3 (tumo?r$ or neoplasm or adenoma$ or carcinoma$)).ti,ab.	8099
15	(carcinoid$ or insulinoma$ or gastrinoma$ or glucagonoma$ or vipoma$ or somatostatinoma$ or apudoma$ or adenoma$ or nesidioblastoma$ or argentaffinoma$).ti,ab.	78273
16	(multiple endocrine adj3 (neoplasia$ or adenopath$ or adenomatos?s or neoplasm$)).ti,ab.	4346
17	((islet or island or beta) adj3 (cell adj2 (tumo?r$ or adenoma$ or carcinoma$))).ti,ab.	2655
18	(familial endocrine adj3 adenomatos?s).ti,ab.	8
19	((diarrheogenic or VIP secreting) adj3 tumo?r$).ti,ab.	39
20	watery diarrh$ syndrome.ti,ab.	58
21	"pancreatic cholera".ti,ab.	59
22	((verner morrison or zollinger ellison) adj3 syndrome).ti,ab.	2086
23	or/14-22	89417
24	13 or 23	152163
25	exp Liver Neoplasms/sc [Secondary]	23534
26	exp Neoplasm Metastasis/	141648
27	exp Liver/	366430

28	(liver or hepatic).ti,ab.	649222
29	27 or 28	759339
30	26 and 29	10571
31	((secondar$ or spread or advanced) adj3 (tumo?r$ or cancer or neoplasm$ or adenoma$ or carcinoma$)).ti,ab.	66995
32	((tumo?r$ or cancer or neoplasm$ or adenoma$ or carcinoma$) adj10 (liver or hepatic)).ti,ab.	68286
33	31 and 32	3532
34	((liver or hepatic) adj3 metasta*).ti,ab.	23506
35	25 or 30 or 33 or 34	40344
36	24 and 35	6016
37	limit 36 to animals	515
38	limit 37 to humans	319
39	37 not 38	196
40	36 not 39	5820
41	limit 40 to "all child (0 to 18 years)"	426
42	limit 41 to "all adult (19 plus years)"	349
43	41 not 42	77
44	40 not 43	5743
45	exp Hepatectomy/	19373
46	exp Liver Neoplasms/sc and exp Liver Neoplasms/su [Secondary,Surgery]	5195
47	((resection or segmentectomy or metastasectomy or surgery) adj5 (liver or hepatic)).ti,ab.	15938
48	45 or 46 or 47	30304
49	exp Chemotherapy, Adjuvant/ or exp Chemoradiotherapy, Adjuvant/ or exp Radiotherapy, Adjuvant/	34831
50	exp Neoadjuvant Therapy/	8305
51	(adjuvan* or neoadjuvan* or neo-adjuvan*).ti,ab.	94382
52	49 or 50 or 51	113432
53	48 and 52	1913
54	exp Disease-Free Survival/ or exp Survival Analysis/ or exp Survival/ or exp Survival Rate/	247981
55	exp "Quality of Life"/	99185
56	(quality adj3 life).ti,ab.	121996
57	surviv*.mp.	798316
58	or/54-57	950694
59	44 and 53	77
60	58 and 59	56

1. **Resection of small bowel adenocarcinoma liver metastasis combined with neoadjuvant and adjuvant chemotherapy results in extended disease-free period-a case report.**

Eigenbrod T. Kullmann F. Klebl F.

International Journal of Gastrointestinal Cancer. 37(2-3):94-7, 2006.
[Journal Article]

UI: 17827529

Authors Full Name

Eigenbrod, Tatjana. Kullmann, Frank. Klebl, Frank.

embase
BIOMEDICAL ANSWERS

Embase Session Results

No.	Query	Results
#18	**#12** AND **#17**	**95**
#17	**#13** OR **#14** OR **#15** OR **#16**	**1,089,046**
#16	**surviv***:ab,ti	**766,227**
#15	(**quality** NEAR/3 **life**):ab,ti	**167,889**
#14	**'quality of life'**/exp	**205,230**
#13	**'survival'**/exp	**456,216**
#12	**#1** AND **#5** AND **#11**	**140**
#11	**#6** OR **#8** OR **#9**	**149,829**
#10	**'neo-adjuvans'**:ab,ti	**2**
#9	**neoadjuvan***:ab,ti	**18,955**
#8	**adjuvan***:ab,ti	**109,916**
#6	**'cancer adjuvant therapy'**/exp OR **'adjuvant chemoradiotherapy'**/exp OR **'adjuvant chemotherapy'**/exp OR **'adjuvant therapy'**/exp	**69,916**
#5	**#2** OR **#3** OR **#4**	**40,937**
#4	**'liver resection'**/exp	**30,103**
#3	((**resection** OR **segmentectomy** OR **metastasectomy** OR **surgery**) NEAR/5 (**liver** OR **hepatic**)):ab,ti	**20,450**
#2	**'liver metastasis'**/exp/dm_su	**5,083**
#1		

No.	Query	Results
#1.43	**#1.39** NOT **#1.42**	**7,295**
#1.42	**#1.40** NOT **#1.41**	
#1.41	**#1.35** NOT **#1.38** AND ([newborn]/lim OR [infant]/lim OR [preschool]/lim OR [school]/lim OR [child]/lim OR [adolescent]/lim) AND ([adult]/lim OR [aged]/lim)	
#1.40	**#1.35** NOT **#1.38** AND ([newborn]/lim OR [infant]/lim OR [preschool]/lim OR [school]/lim OR [child]/lim OR [adolescent]/lim)	

**#1.39**	**#1.35** NOT **#1.38**
**#1.38**	**#1.36** NOT **#1.37**
**#1.37**	**#1.28** AND **#1.34** AND [animals]/lim AND [humans]/lim
**#1.36**	**#1.28** AND **#1.34** AND [animals]/lim
**#1.35**	**#1.28** AND **#1.34**
**#1.34**	**#1.29** OR **#1.32** OR **#1.33**
**#1.33**	(**metasta*** NEAR/3 (**liver** OR **hepatic**)):ab,ti
**#1.32**	**#1.30** AND **#1.31**
**#1.31**	((**tumor*** OR **tumour*** OR **cancer** OR **neoplasm*** OR **adenoma*** OR **carcinoma***) NEAR/10 (**liver** OR **hepatic**)):ab,ti
**#1.30**	((**secondar*** OR **spread** OR **advanced**) NEAR/3 (**tumor*** OR **tumour*** OR **cancer** OR **neoplasm*** OR **adenoma*** OR **carcinoma***)):ab,ti
**#1.29**	**'liver metastasis'**/exp
**#1.28**	**#1.13** OR **#1.27**
**#1.27**	**#1.14** OR **#1.15** OR **#1.16** OR **#1.19** OR **#1.20** OR **#1.21** OR **#1.24** OR **#1.25** OR **#1.26**
**#1.26**	((**'verner morrison'** OR **'zollinger ellison'**) NEAR/3 **syndrome**):ab,ti
**#1.25**	**'pancreatic cholera'**:ab,ti
**#1.24**	**#1.22** AND **#1.23**
**#1.23**	(**diarr*** NEAR/3 **syndrome**):ab,ti
**#1.22**	(**watery** NEAR/3 **diarr***):ab,ti
**#1.21**	((**diarrheogenic** OR **'vip secreting'** OR **'vip secreting'**) NEAR/3 (**tumor*** OR **tumour***)):ab,ti
**#1.20**	**familial**:ab,ti AND (**endocrine** NEAR/3 **adenomatos?s**):ab,ti
**#1.19**	**#1.17** AND **#1.18**
**#1.18**	((**islet** OR **island** OR **beta**) NEAR/3 **cell**):ab,ti
**#1.17**	(**cell** NEAR/2 (**tumor*** OR **tumour*** OR **adenoma*** OR **carcinoma***)):ab,ti
**#1.16**	**multiple**:ab,ti AND (**endocrine** NEAR/3 (**neoplasia*** OR **adenopath*** OR **adenomatos?s** OR **neoplasm***)):ab,ti
**#1.15**	**carcinoid***:ab,ti OR **insulinoma***:ab,ti OR **gastrinoma***:ab,ti OR **glucagonoma***:ab,ti OR **vipoma***:ab,ti OR **somatostatinoma***:ab,ti OR **apudoma***:ab,ti OR **adenoma***:ab,ti OR **nesidioblastoma***:ab,ti OR **argentaffinoma***:ab,ti
**#1.14**	((**neuroendocrine** OR **gastroenteropancreatic**) NEAR/3 (**tumor*** OR **tumour*** OR **neoplasm** OR **adenoma*** OR **carcinoma***)):ab,ti
**#1.13**	**#1.1** OR **#1.2** OR **#1.3** OR **#1.4** OR **#1.5** OR **#1.6** OR **#1.7** OR **#1.8** OR **#1.9** OR **#1.10** OR **#1.11** OR **#1.12**
**#1.12**	**'pancreas tumor'**/exp
**#1.11**	**'multiple endocrine neoplasia'**/exp

#1.10	**'vipoma'**/exp
#1.9	**'somatostatinoma'**/exp
#1.8	**'glucagonoma'**/exp
#1.7	**'gastrinoma'**/exp
#1.6	**'pancreas islet cell carcinoma'**/exp
#1.5	**'insulinoma'**/exp
#1.4	**'pancreas islet cell tumor'**/exp
#1.3	**'carcinoid'**/exp
#1.2	**'apudoma'**/exp
#1.1	**'neuroendocrine tumor'**/exp

ELSEVIER Copyright © 2012 Elsevier B.V. All rights reserved. Embase is a trademark of Elsevier B.V.

Search Name: NET-Session_11

Comments: Breitenstein USZ

Save Date: 2012-05-27 07:00:06.266

ID	Search
#1	MeSH descriptor Neuroendocrine Tumors explode all trees
#2	MeSH descriptor Apudoma explode all trees
#3	MeSH descriptor Carcinoid Tumor explode all trees
#4	MeSH descriptor Adenoma, Islet Cell explode all trees
#5	MeSH descriptor Insulinoma explode all trees
#6	MeSH descriptor Carcinoma, Islet Cell explode all trees
#7	MeSH descriptor Gastrinoma explode all trees
#8	MeSH descriptor Glucagonoma explode all trees
#9	MeSH descriptor Somatostatinoma explode all trees
#10	MeSH descriptor Vipoma explode all trees
#11	MeSH descriptor Multiple Endocrine Neoplasia explode all trees
#12	MeSH descriptor Pancreatic Neoplasms explode all trees
#13	(#1 OR #2 OR #3 OR #4 OR #5 OR #6 OR #7 OR #8 OR #9 OR (#10 AND #11) OR #12)
#14	((neuroendocrine OR gastroenteropancreatic) NEAR/3 (tumor* OR tumour* OR neoplasm OR adenoma* OR carcinoma*)):ti,ab,kw or (carcinoid* or insulinoma* or gastrinoma* or glucagonoma* or vipoma* or somatostatinoma* or apudoma* or adenoma* or nesidioblastoma* or argentaffinoma*):ti,ab,kw or ((multiple endocrine) NEAR/3 (neoplasia* OR adenopath* OR adenomatos?s OR neoplasm*)):ti,ab,kw
#15	((islet or island or beta) NEAR/3 (cell NEAR/2 (tumo?r* or adenoma* or carcinoma*))):ti,ab,kw
#16	((familial endocrine) NEAR/3 adenomatos?s):ti,ab,kw
#17	((diarrheogenic OR 'vip secreting' OR 'vip secreting') NEAR/3 (tumor* OR tumour*)):ti,ab,kw
#18	(watery diarrh$ syndrome):ti,ab,kw
#19	"pancreatic cholera":ti,ab,kw
#20	(('verner morrison' OR 'zollinger ellison') NEAR/3 syndrome):ti,ab,kw
#21	(#14 OR #15)
#22	(#13 OR #21)
#23	MeSH descriptor Neoplasm Metastasis explode all trees
#24	MeSH descriptor Liver explode all trees
#25	(liver or hepatic):ti or (liver or hepatic):ab
#26	(#24 OR #25)
#27	(#23 AND #26)
#28	((secondar* OR spread OR advanced) NEAR/3 (tumor* OR tumour* OR cancer OR neoplasm* OR adenoma* carcinoma*)):ti,ab,kw

#29 ((tumor* OR tumour* OR cancer OR neoplasm* OR adenoma* OR carcinoma*) NEAR/10 (liver OR hepatic)):ti,ab,kw

#30 (#28 AND #29)

#31 (metasta* NEAR/3 (liver OR hepatic)):ti,ab,kw

#32 MeSH descriptor Liver Neoplasms explode all trees with qualifier: SC

#33 (#27 OR #30 OR #31 OR #32)

#34 (#22 AND #33)

#35 MeSH descriptor Melanoma explode all trees

#36 MeSH descriptor Neurilemma explode all trees

#37 (#36 OR #35)

#38 (#34 AND NOT #37)

#39 MeSH descriptor Hepatectomy explode all trees

#40 MeSH descriptor Liver Neoplasms explode all trees with qualifier: SC

#41 MeSH descriptor Liver Neoplasms explode all trees with qualifier: SU

#42 (#40 AND #41)

#43 ((resection OR segmentectomy OR metastasectomy OR surgery) NEAR/5 (liver OR hepatic)):ti,ab,kw

#44 (#39 OR #42 OR #43)

#45 MeSH descriptor Survival Rate explode all trees

#46 MeSH descriptor Survival Analysis explode all trees

#47 MeSH descriptor Disease-Free Survival explode all trees

#48 MeSH descriptor Quality of Life explode all trees

#49 (quality NEAR/3 life):ti,ab,kw or (surviv*):ti,ab,kw

#50 (#45 OR #46 OR #47 OR #48 OR #49)

#51 MeSH descriptor Chemotherapy, Adjuvant explode all trees

#52 MeSH descriptor Chemoradiotherapy, Adjuvant explode all trees

#53 MeSH descriptor Radiotherapy, Adjuvant explode all trees

#54 MeSH descriptor Neoadjuvant Therapy explode all trees

#55 (adjuvan* or neoadjuvan* or neo-adjuvan*):ti,ab,kw

#56 (#51 OR #52 OR #53 OR #54 OR #55)

#57 (#38 AND #44 AND #50 AND #56)

ID	Search	Hits	Edit	Delete
#1	MeSH descriptor **Neuroendocrine Tumors** explode all trees	1156	edit	delete
#2	MeSH descriptor **Apudoma** explode all trees	0	edit	delete
#3	MeSH descriptor **Carcinoid Tumor** explode all trees	44	edit	delete
#4	MeSH descriptor **Adenoma, Islet Cell** explode all trees	15	edit	delete
#5	MeSH descriptor **Insulinoma** explode all trees	9	edit	delete
#6	MeSH descriptor **Carcinoma, Islet Cell** explode all trees	6	edit	delete
#7	MeSH descriptor **Gastrinoma** explode all trees	3	edit	delete
#8	MeSH descriptor **Glucagonoma** explode all trees	0	edit	delete
#9	MeSH descriptor **Somatostatinoma** explode all trees	1	edit	delete
#10	MeSH descriptor **Vipoma** explode all trees	2	edit	delete
#11	MeSH descriptor **Multiple Endocrine Neoplasia** explode all trees	7	edit	delete
#12	MeSH descriptor **Pancreatic Neoplasms** explode all trees	690	edit	delete
#13	(#1 OR #2 OR #3 OR #4 OR #5 OR #6 OR #7 OR #8 OR #9 OR (#10 AND #11) OR #12)	1822	edit	delete
#14	((neuroendocrine OR gastroenteropancreatic) NEAR/3 (tumor* OR tumour* OR neoplasm OR adenoma* OR carcinoma*)):ti,ab,kw or (carcinoid* or insulinoma* or gastrinoma* or glucagonoma* or vipoma* or somatostatinoma* or apudoma* or adenoma* or nesidioblastoma* or argentaffinoma*):ti,ab,kw or ((multiple endocrine) NEAR/3 (neoplasia* OR adenopath* OR adenomatos?s OR neoplasm*)):ti,ab,kw	1304	edit	delete
#15	((islet or island or beta) NEAR/3 (cell NEAR/2 (tumo?r* or adenoma* or carcinoma*))):ti,ab,kw	15	edit	delete
#16	((familial endocrine) NEAR/3 adenomatos?s):ti,ab,kw	0	edit	delete
#17	((diarrheogenic OR 'vip secreting' OR 'vip secreting') NEAR/3 (tumor* OR tumour*)):ti,ab,kw	0	edit	delete
#18	(watery diarrh$ syndrome):ti,ab,kw	0	edit	delete
#19	"pancreatic cholera":ti,ab,kw	0	edit	delete
#20	(('verner morrison' OR 'zollinger ellison') NEAR/3 syndrome):ti,ab,kw	0	edit	delete
#21	(#14 OR #15)	1310	edit	delete
#22	(#13 OR #21)	3010	edit	delete
#23	MeSH descriptor **Neoplasm Metastasis** explode all trees	3275	edit	delete
#24	MeSH descriptor **Liver** explode all trees	2339	edit	delete
#25	(liver or hepatic):ti or (liver or hepatic):ab	24742	edit	delete
#26	(#24 OR #25)	25211	edit	delete
#27	(#23 AND #26)	185	edit	delete
#28	((secondar* OR spread OR advanced) NEAR/3 (tumor* OR tumour* OR cancer OR neoplasm* OR adenoma* carcinoma*)):ti,ab,kw	7713	edit	delete
#29	((tumor* OR tumour* OR cancer OR neoplasm* OR adenoma* OR carcinoma*) NEAR/10 (liver OR hepatic)):ti,ab,kw	2779	edit	delete
#30	(#28 AND #29)	612	edit	delete
#31	(metasta* NEAR/3 (liver OR hepatic)):ti,ab,kw	791	edit	delete
#32	MeSH descriptor **Liver Neoplasms** explode all trees with qualifier: **SC**	530	edit	delete
#33	(#27 OR #30 OR #31 OR #32)	1219	edit	delete
#34	(#22 AND #33)	101	edit	delete
#35	MeSH descriptor **Melanoma** explode all trees	952	edit	delete
#36	MeSH descriptor **Neurilemma** explode all trees	1	edit	delete
#37	(#36 OR #35)	954	edit	delete
#38	(#34 AND NOT #37)	69	edit	delete
#39	MeSH descriptor **Hepatectomy** explode all trees	404	edit	delete
#40	MeSH descriptor **Liver Neoplasms** explode all trees with qualifier: **SC**	530	edit	delete
#41	MeSH descriptor **Liver Neoplasms** explode all trees with qualifier: **SU**	410	edit	delete
#42	(**#40** AND **#41**)	99	edit	delete
#43	((resection OR segmentectomy OR metastasectomy OR surgery) NEAR/5 (liver OR hepatic)):ti,ab,kw	1345	edit	delete
#44	(**#39** OR **#42** OR **#43**)	1409	edit	delete
#45	MeSH descriptor **Survival Rate** explode all trees	7198	edit	delete
#46	MeSH descriptor **Survival Analysis** explode all trees	12895	edit	delete
#47	MeSH descriptor **Disease-Free Survival** explode all trees	3635	edit	delete
#48	MeSH descriptor **Quality of Life** explode all trees	11889	edit	delete
#49	(quality NEAR/3 life):ti,ab,kw or (surviv*):ti,ab,kw	56737	edit	delete
#50	(**#45** OR **#46** OR **#47** OR **#48** OR **#49**)	58558	edit	delete

#51	MeSH descriptor **Chemotherapy, Adjuvant** explode all trees	2917	edit	delete
#52	MeSH descriptor **Chemoradiotherapy, Adjuvant** explode all trees	2	edit	delete
#53	MeSH descriptor **Radiotherapy, Adjuvant** explode all trees	779	edit	delete
#54	MeSH descriptor **Neoadjuvant Therapy** explode all trees	549	edit	delete
#55	(adjuvan* or neoadjuvan* or neo-adjuvan*):ti,ab,kw	13756	edit	delete
#56	(#51 OR #52 OR #53 OR #54 OR #55)	13756	edit	delete
#57	(#38 AND #44 AND #50 AND #56)	3	edit	delete
