# Supplementary material for: Transplantation and Surgical Strategies in Patients With Neuroendocrine Liver Metastases: Protocol of Four Systematic Reviews
Source: JMIR Res Protoc. 2013 Dec 23;2(2):e58. doi: 10.2196/resprot.2891 (PMC3875902; doi:10.2196/resprot.2891)
Supplement: Supplementary file 2 [file resprot_v2i2e58_app2.pdf]

CONSORT-EHEALTH Checklist V1.6.2 Report

(based on CONSORT-EHEALTH V1.6), available at [http://tinyurl.com/consort-ehealth-v1-6].

2891

Date completed

10/6/2013 8:19:47

by

Stefan Breitenstein

Transplant and Surgical Strategies in Patients with NET Liver Metastases – A Protocol of Four Systematic Reviews

TITLE

1a-i) Identify the mode of delivery in the title

The manuscript submitted is a protocol for a systematic review, therefore not applicable.

1a-ii) Non-web-based components or important co-interventions in title

The manuscript submitted is a protocol for a systematic review, therefore not applicable.

1a-iii) Primary condition or target group in the title

Transplant and Surgical Strategies in Patients with Neuroendocrine Liver Metastases

ABSTRACT

1b-i) Key features/functionalities/components of the intervention and comparator in the METHODS section of the ABSTRACT

The manuscript submitted is a protocol for a systematic review, therefore not applicable.

1b-ii) Level of human involvement in the METHODS section of the ABSTRACT

The manuscript submitted is a protocol for a systematic review, therefore not applicable.

1b-iii) Open vs. closed, web-based (self-assessment) vs. face-to-face assessments in the METHODS section of the ABSTRACT

The manuscript submitted is a protocol for a systematic review, therefore not applicable.

1b-iv) RESULTS section in abstract must contain use data

The manuscript submitted is a protocol for a systematic review, therefore not applicable.

1b-v) CONCLUSIONS/DISCUSSION in abstract for negative trials

The manuscript submitted is a protocol for a systematic review, therefore not applicable.

INTRODUCTION

2a-i) Problem and the type of system/solution

Neuroendocrine tumours:
Neuroendocrine tumours (NETs) derive from neuroendocrine cells and can therefore arise almost everywhere in the entire body [1]. Primary NETs are mainly located in the bronchopulmonary (>25%) and the gastroenteropancreatic system (60%) [2, 3]. With an incidence of 5.25 per 100’000 per year NETs are considered to be rare tumours. Most NETs occur sporadically, while a minority develops due to a genetic background found in patients suffering from syndromes such as multiple endocrine neoplasia type 1 (MEN1) [4].

NETs are subdivided in two groups according to their functional behaviour [5]. Functioning NETs secrete biogenic amines as well as polypeptide hormones and can cause endocrine syndromes such as the carcinoid-syndrome. Endocrine syndromes in tumours with portal venous drainage often begin in the presence of liver metastases. Metastases drain active hormones directly into the systematic circulation as a result of liver metabolises hormones deriving from primary tumours [6, 7]. Therefore, functioning tumours are usually detected earlier than non-functioning tumours and patients seem to have a better overall survival (OS) [8]. The non-functioning NETs may cause local mass related symptoms or are found incidentally [7, 9].

Liver metastases of NETs:
Despite the slow growing nature of NETs, Pape et al. reported liver metastases of gastroenteropancreatic NETs present at initial diagnosis in 84.7% of cases [10]. Due to the favourable environment, metastases of NETs are confined to the liver for a prolonged period of time [11]. Hepatic metastases are considered to be a major prognostic factor, associated with a significantly reduced survival compared to patients without liver metastases [12, 13]. Furthermore, the metastatic pattern within the liver also has prognostic and therapeutic impact. Frilling et al. suggested a classification in three different types of liver metastases: “single metastasis of any size (type 1); isolated metastatic bulk accompanied by smaller deposits, with both liver lobes always involved (type 2); and disseminated metastatic spread, with both liver lobes always involved, single lesion of varying size and virtually no normal liver parenchyma (type 3).” This classification is believed to represent differences in biological behaviour of the tumours, which require different treatment strategies [14].

Liver resection:
A wide array of options is available to treat liver metastases from NETs, which improves the 5-year OS from <40% in untreated patients to 60-80% in patients who have undergone curative surgery [15-17]. Surgical interventions contain potentially curative resection of the metastases (R0/R1). If R0/R1 resection is not feasible, a palliative resection is indicated in patients suffering from tumour bulk or hormonal symptoms, especially in patients with functioning NETs and who are unresponsive to treatment. However, guidelines suggest that palliative surgery should only be performed if at least 90% of the metastatic bulk can be removed [18]. Curative resection can only be achieved in patients with a metastatic pattern type 1, while patients with type 2 or 3 need to be evaluated for other treatment options [14]. Therefore curative resection is only feasible in maximum 20% of patients due to the high rate of diffuse and bilobar metastatic spread [19]. For patients with a metastatic pattern type 2 or 3, there are several loco-regional techniques such as radiofrequency ablation (RFA) or transcatheter arterial chemoembolisation (TACE), or systemically applied therapies such as chemotherapy or peptide receptor radionuclide therapy (PRRT) [17]. The first systematic review intends to compare curative and palliative liver resection versus or in combination with non-surgical treatment options.

Neoadjuvant and adjuvant treatment options:
Disease recurrence after surgical treatment of liver metastases is often observed, even when resection is performed with curative intent [20]. In order to increase resectability and to reduce the high rate of metastatic relapse, neoadjuvant and adjuvant treatment options need to be evaluated. According to their treatment modality, neoadjuvant und adjuvant treatment options can be divided into systemic (chemotherapy, biotherapy and PRRT) and liver-directed therapies (selective internal radiation therapy (SIRT), transcatheter arterial embolisation/chemoembolisation (TAE/TACE)). For the chemotherapeutical strategy, several substances have been used to treat NETs, either as a monotherapy or combined in different regimens [21-23]. Biotherapy essentially includes somatostatin analogues, such as octreotide and lanreotide, in order to control hormone-related symptoms [24]. PRRT consists of systemically applied radiolabeled somatostatin derivates that bind specifically to the somatostatin receptor which is overexpressed in certain NETs and thereby damage the tumour cell [25]. Liver directed techniques, such as SIRT, TAE and TACE, make use of the biologic feature that hepatic neoplasms are preferentially supplied via the hepatic artery, whereas normal liver parenchyma is mainly supplied by the portal vein [26, 27].
For liver metastases arising from a non-NET primary tumour, the benefit of neoadjuvant and adjuvant strategies combined with liver resection has already been investigated in more detail [28, 29]. Nordlinger et al. showed that the risk of recurrent disease in patients with liver metastases of colorectal carcinomas could be reduced compared to surgical resections alone [29]. Adopting these strategies to the treatment of NET liver metastasis could be a promising option. The second systematic review intends to evaluate, if neoadjuvant and/or adjuvant treatment strategies together with surgical resection is superior to liver resection alone.

Liver transplantation:
Controversy concerning the indication for liver transplantations arises due to the relatively low number performed in patients with liver metastases from NETs. Moreover, heterogeneous 5-year OS data have been published with ranges from 33% to 96% [16, 30]. Therefore, our third systematic review aims to evaluate the possible benefit of liver transplantation as a treatment option for unresectable hepatic metastases of NETs and to define selection criteria to choose patients with the best possible prognosis.

Resection of the primary NET:
Another important question is whether the primary tumour should be removed in presence of non-resectable liver metastases as the answer may improve the outcome. Potential benefits are seen in providing relief from hormonal and local tumor mass-related symptoms [31]. Since evidence is missing, the fourth systematic review aims to answer this question.

The aim of these four systematic reviews is to assess the role of surgical strategies in the management of liver metastases of NET’s, to evaluate the use of adjuvant and neoadjuvant therapies, to define selection criteria for patients benefit the most from liver transplantation and to study the influence of resection of the primary tumour.

2a-ii) Scientific background, rationale: What is known about the (type of) system

Neuroendocrine tumours:
Neuroendocrine tumours (NETs) derive from neuroendocrine cells and can therefore arise almost everywhere in the entire body [1]. Primary NETs are mainly located in the bronchopulmonary (>25%) and the gastroenteropancreatic system (60%) [2, 3]. With an incidence of 5.25 per 100'000 per year NETs are considered to be rare tumours. Most NETs occur sporadically, while a minority develops due to a genetic background found in patients suffering from syndromes such as multiple endocrine neoplasia type 1 (MEN1) [4].

NETs are subdivided in two groups according to their functional behaviour [5]. Functioning NETs secrete biogenic amines as well as polypeptide hormones and can cause endocrine syndromes such as the carcinoid-syndrome. Endocrine syndromes in tumours with portal venous drainage often begin in the presence of liver metastases. Metastases drain active hormones directly into the systematic circulation as a result of liver metabolises hormones deriving from primary tumours [6, 7]. Therefore, functioning tumours are usually detected earlier than non-functioning tumours and patients seem to have a better overall survival (OS) [8]. The non-functioning NETs may cause local mass related symptoms or are found incidentally [7, 9].

Liver metastases of NETs:
Despite the slow growing nature of NETs, Pape et al. reported liver metastases of gastroenteropancreatic NETs present at initial diagnosis in 84.7% of cases [10]. Due to the favourable environment, metastases of NETs are confined to the liver for a prolonged period of time [11]. Hepatic metastases are considered to be a major prognostic factor, associated with a significantly reduced survival compared to patients without liver metastases [12, 13]. Furthermore, the metastatic pattern within the liver also has prognostic and therapeutic impact. Frilling et al. suggested a classification in three different types of liver metastases: "single metastasis of any size (type 1); isolated metastatic bulk accompanied by smaller deposits, with both liver lobes always involved (type 2); and disseminated metastatic spread, with both liver lobes always involved, single lesion of varying size and virtually no normal liver parenchyma (type 3)." This classification is believed to represent differences in biological behaviour of the tumours, which require different treatment strategies [14].

Liver resection:
A wide array of options is available to treat liver metastases from NETs, which improves the 5-year OS from <40% in untreated patients to 60-80% in patients who have undergone curative surgery [15-17]. Surgical interventions contain potentially curative resection of the metastases (R0/R1). If R0/R1 resection is not feasible, a palliative resection is indicated in patients suffering from tumour bulk or hormonal symptoms, especially in patients with functioning NETs and who are unresponsive to treatment. However, guidelines suggest that palliative surgery should only be performed if at least 90% of the metastatic bulk can be removed [18]. Curative resection can only be achieved in patients with a metastatic pattern type 1, while patients with type 2 or 3 need to be evaluated for other treatment options [14]. Therefore curative resection is only feasible in maximum 20% of patients due to the high rate of diffuse and bilobar metastatic spread [19]. For patients with a metastatic pattern type 2 or 3, there are several loco-regional techniques such as radiofrequency ablation (RFA) or transcatheter arterial chemoembolisation (TACE), or systemically applied therapies such as chemotherapy or peptide receptor radionuclide therapy (PRRT) [17]. The first systematic review intends to compare curative and palliative liver resection versus or in combination with non-surgical treatment options.

Neoadjuvant and adjuvant treatment options:
Disease recurrence after surgical treatment of liver metastases is often observed, even when resection is performed with curative intent [20]. In order to increase resectability and to reduce the high rate of metastatic relapse, neoadjuvant and adjuvant treatment options need to be evaluated. According to their treatment modality, neoadjuvant und adjuvant treatment options can be divided into systemic (chemotherapy, biotherapy and PRRT) and liver-directed therapies (selective internal radiation therapy (SIRT), transcatheter arterial embolisation/chemoembolisation (TAE/TACE)). For the chemotherapeutical strategy, several substances have been used to treat NETs, either as a monotherapy or combined in different regimens [21-23]. Biotherapy essentially includes somatostatin analogues, such as octreotide and lanreotide, in order to control hormone-related symptoms [24]. PRRT consists of systemically applied radiolabeled somatostatin derivates that bind specifically to the somatostatin receptor which is overexpressed in certain NETs and thereby damage the tumour cell [25]. Liver directed techniques, such as SIRT, TAE and TACE, make use of the biologic feature that hepatic neoplasms are preferentially supplied via the hepatic artery, whereas normal liver parenchyma is mainly supplied by the portal vein [26, 27].
For liver metastases arising from a non-NET primary tumour, the benefit of neoadjuvant and adjuvant strategies combined with liver resection has already been investigated in more detail [28, 29]. Nordlinger et al. showed that the risk of recurrent disease in patients with liver metastases of colorectal carcinomas could be reduced compared to surgical resections alone [29]. Adopting these strategies to the treatment of NET liver metastasis could be a promising option. The second systematic review intends to evaluate, if neoadjuvant and/or adjuvant treatment strategies together with surgical resection is superior to liver resection alone.

Liver transplantation:
Controversy concerning the indication for liver transplantations arises due to the relatively low number performed in patients with liver metastases from NETs. Moreover, heterogeneous 5-year OS data have been published with ranges from 33% to 96% [16, 30]. Therefore, our third systematic review aims to evaluate the possible benefit of liver transplantation as a treatment option for unresectable hepatic metastases of NETs and to define selection criteria to choose patients with the best possible prognosis.

Resection of the primary NET:
Another important question is whether the primary tumour should be removed in presence of non-resectable liver metastases as the answer may improve the outcome. Potential benefits are seen in providing relief from hormonal and local tumor mass-related symptoms [31]. Since evidence is missing, the fourth systematic review aims to answer this question.

The aim of these four systematic reviews is to assess the role of surgical strategies in the management of liver metastases of NET's, to evaluate the use of adjuvant and neoadjuvant therapies, to define selection criteria for patients benefit the most from liver transplantation and to study the influence of resection of the primary tumour.

METHODS

3a) CONSORT: Description of trial design (such as parallel, factorial) including allocation ratio

The manuscript submitted is a protocol for a systematic review, therefore not applicable.

3b) CONSORT: Important changes to methods after trial commencement (such as eligibility criteria), with reasons

The manuscript submitted is a protocol for a systematic review, therefore not applicable.

3b-i) Bug fixes, Downtimes, Content Changes

The manuscript submitted is a protocol for a systematic review, therefore not applicable.

4a) CONSORT: Eligibility criteria for participants

The manuscript submitted is a protocol for a systematic review, therefore not applicable. Literature search was neither restricted by language nor by publication date. All accessible publications were included. The following study designs were included for the qualitative synthesis of the systematic review: Randomised Controlled Trials (RCTs), prospective and retrospective comparative cohort studies and case-control studies. Case series were only included in a separate database for descriptive purposes.

4a-i) Computer / Internet literacy

The manuscript submitted is a protocol for a systematic review, therefore not applicable.
4a-ii) Open vs. closed, web-based vs. face-to-face assessments:
The manuscript submitted is a protocol for a systematic review, therefore not applicable.
4a-iii) Information giving during recruitment
The manuscript submitted is a protocol for a systematic review, therefore not applicable.
4b) CONSORT: Settings and locations where the data were collected
The manuscript submitted is a protocol for a systematic review, therefore not applicable.
4b-i) Report if outcomes were (self-)assessed through online questionnaires
The manuscript submitted is a protocol for a systematic review, therefore not applicable.
4b-ii) Report how institutional affiliations are displayed
The manuscript submitted is a protocol for a systematic review, therefore not applicable.
5) CONSORT: Describe the interventions for each group with sufficient details to allow replication, including how and when they were actually administered
5-i) Mention names, credential, affiliations of the developers, sponsors, and owners
The manuscript submitted is a protocol for a systematic review, therefore not applicable.
5-ii) Describe the history/development process
The manuscript submitted is a protocol for a systematic review, therefore not applicable.
5-iii) Revisions and updating
The manuscript submitted is a protocol for a systematic review, therefore not applicable.
5-iv) Quality assurance methods
The manuscript submitted is a protocol for a systematic review, therefore not applicable.
5-v) Ensure replicability by publishing the source code, and/or providing screenshots/screen-capture video, and/or providing flowcharts of the algorithms used
The manuscript submitted is a protocol for a systematic review, therefore not applicable.
5-vi) Digital preservation
The manuscript submitted is a protocol for a systematic review, therefore not applicable.
5-vii) Access
The manuscript submitted is a protocol for a systematic review, therefore not applicable.
5-viii) Mode of delivery, features/functionalities/components of the intervention and comparator, and the theoretical framework
The manuscript submitted is a protocol for a systematic review, therefore not applicable.
5-ix) Describe use parameters
The manuscript submitted is a protocol for a systematic review, therefore not applicable.
5-x) Clarify the level of human involvement
The manuscript submitted is a protocol for a systematic review, therefore not applicable.
5-xi) Report any prompts/reminders used
The manuscript submitted is a protocol for a systematic review, therefore not applicable.
5-xii) Describe any co-interventions (incl. training/support)
The manuscript submitted is a protocol for a systematic review, therefore not applicable.
6a) CONSORT: Completely defined pre-specified primary and secondary outcome measures, including how and when they were assessed
The manuscript submitted is a protocol for a systematic review, therefore not applicable.
6a-i) Online questionnaires: describe if they were validated for online use and apply CHERRIES items to describe how the questionnaires were designed/deployed
The manuscript submitted is a protocol for a systematic review, therefore not applicable.
6a-ii) Describe whether and how “use” (including intensity of use/dosage) was defined/measured/monitored
The manuscript submitted is a protocol for a systematic review, therefore not applicable.
6a-iii) Describe whether, how, and when qualitative feedback from participants was obtained
The manuscript submitted is a protocol for a systematic review, therefore not applicable.
6b) CONSORT: Any changes to trial outcomes after the trial commenced, with reasons
The manuscript submitted is a protocol for a systematic review, therefore not applicable.
7a) CONSORT: How sample size was determined
7a-i) Describe whether and how expected attrition was taken into account when calculating the sample size
The manuscript submitted is a protocol for a systematic review, therefore not applicable.
7b) CONSORT: When applicable, explanation of any interim analyses and stopping guidelines
The manuscript submitted is a protocol for a systematic review, therefore not applicable.
8a) CONSORT: Method used to generate the random allocation sequence
The manuscript submitted is a protocol for a systematic review, therefore not applicable.
8b) CONSORT: Type of randomisation; details of any restriction (such as blocking and block size)
The manuscript submitted is a protocol for a systematic review, therefore not applicable.
9) CONSORT: Mechanism used to implement the random allocation sequence (such as sequentially numbered containers), describing any steps taken to conceal the sequence until interventions were assigned
The manuscript submitted is a protocol for a systematic review, therefore not applicable.
10) CONSORT: Who generated the random allocation sequence, who enrolled participants, and who assigned participants to interventions
The manuscript submitted is a protocol for a systematic review, therefore not applicable.
11a) CONSORT: Blinding - If done, who was blinded after assignment to interventions (for example, participants, care providers, those assessing outcomes) and how
11a-i) Specify who was blinded, and who wasn’t
The manuscript submitted is a protocol for a systematic review, therefore not applicable.
11a-ii) Discuss e.g., whether participants knew which intervention was the “intervention of interest” and which one was the “comparator”
The manuscript submitted is a protocol for a systematic review, therefore not applicable.
11b) CONSORT: If relevant, description of the similarity of interventions
The manuscript submitted is a protocol for a systematic review, therefore not applicable.

12a) CONSORT: Statistical methods used to compare groups for primary and secondary outcomes
The manuscript submitted is a protocol for a systematic review, therefore not applicable.
12a-i) Imputation techniques to deal with attrition / missing values
The manuscript submitted is a protocol for a systematic review, therefore not applicable.
12b) CONSORT: Methods for additional analyses, such as subgroup analyses and adjusted analyses
The manuscript submitted is a protocol for a systematic review, therefore not applicable.

RESULTS

13a) CONSORT: For each group, the numbers of participants who were randomly assigned, received intended treatment, and were analysed for the primary outcome
The manuscript submitted is a protocol for a systematic review, therefore not applicable.
13b) CONSORT: For each group, losses and exclusions after randomisation, together with reasons
The manuscript submitted is a protocol for a systematic review, therefore not applicable.
13b-i) Attrition diagram
The manuscript submitted is a protocol for a systematic review, therefore not applicable.
14a) CONSORT: Dates defining the periods of recruitment and follow-up
The manuscript submitted is a protocol for a systematic review, therefore not applicable.
14a-i) Indicate if critical "secular events" fell into the study period
The manuscript submitted is a protocol for a systematic review, therefore not applicable.
14b) CONSORT: Why the trial ended or was stopped (early)
The manuscript submitted is a protocol for a systematic review, therefore not applicable.
15) CONSORT: A table showing baseline demographic and clinical characteristics for each group
The manuscript submitted is a protocol for a systematic review, therefore not applicable.
15-i) Report demographics associated with digital divide issues
The manuscript submitted is a protocol for a systematic review, therefore not applicable.
16a) CONSORT: For each group, number of participants (denominator) included in each analysis and whether the analysis was by original assigned groups
16-i) Report multiple "denominators" and provide definitions
The manuscript submitted is a protocol for a systematic review, therefore not applicable.
16-ii) Primary analysis should be intent-to-treat
The manuscript submitted is a protocol for a systematic review, therefore not applicable.
17a) CONSORT: For each primary and secondary outcome, results for each group, and the estimated effect size and its precision (such as 95% confidence interval)
The manuscript submitted is a protocol for a systematic review, therefore not applicable.
17a-i) Presentation of process outcomes such as metrics of use and intensity of use
The manuscript submitted is a protocol for a systematic review, therefore not applicable.
17b) CONSORT: For binary outcomes, presentation of both absolute and relative effect sizes is recommended
The manuscript submitted is a protocol for a systematic review, therefore not applicable.
18) CONSORT: Results of any other analyses performed, including subgroup analyses and adjusted analyses, distinguishing pre-specified from exploratory
The manuscript submitted is a protocol for a systematic review, therefore not applicable.
18-i) Subgroup analysis of comparing only users
The manuscript submitted is a protocol for a systematic review, therefore not applicable.
19) CONSORT: All important harms or unintended effects in each group
The manuscript submitted is a protocol for a systematic review, therefore not applicable.
19-i) Include privacy breaches, technical problems
The manuscript submitted is a protocol for a systematic review, therefore not applicable.
19-ii) Include qualitative feedback from participants or observations from staff/researchers
The manuscript submitted is a protocol for a systematic review, therefore not applicable.

DISCUSSION

20) CONSORT: Trial limitations, addressing sources of potential bias, imprecision, multiplicity of analyses
20-i) Typical limitations in ehealth trials
The manuscript submitted is a protocol for a systematic review, therefore not applicable. The use of surgical strategies for the treatment of patients with liver metastases from NET is still controversial. An important step towards developing a consensus is to summarize the existing scientific literature. The four systematic reviews described in this protocol will help to elucidate the role of surgical strategies and serve as a basis for developing clinical practice guidelines.
21) CONSORT: Generalisability (external validity, applicability) of the trial findings
21-i) Generalizability to other populations
The manuscript submitted is a protocol for a systematic review, therefore not applicable.
21-ii) Discuss if there were elements in the RCT that would be different in a routine application setting
The manuscript submitted is a protocol for a systematic review, therefore not applicable.
22) CONSORT: Interpretation consistent with results, balancing benefits and harms, and considering other relevant evidence
22-i) Restate study questions and summarize the answers suggested by the data, starting with primary outcomes and process outcomes (use)
The manuscript submitted is a protocol for a systematic review, therefore not applicable.
22-ii) Highlight unanswered new questions, suggest future research
The manuscript submitted is a protocol for a systematic review, therefore not applicable.

Other information

23) CONSORT: Registration number and name of trial registry
International Prospective Register of Systematic Reviews (PROSPERO): CRD42012002652, CRD42012002656, CRD42012002655, and CRD42012002654.
24) CONSORT: Where the full trial protocol can be accessed, if available
International Prospective Register of Systematic Reviews (PROSPERO): CRD42012002652, CRD42012002656, CRD42012002655, and CRD42012002654.

25) CONSORT: Sources of funding and other support (such as supply of drugs), role of funders
No funding.
X26-i) Comment on ethics committee approval
The manuscript submitted is a protocol for a systematic review, therefore not applicable.
x26-ii) Outline informed consent procedures
The manuscript submitted is a protocol for a systematic review, therefore not applicable.
X26-iii) Safety and security procedures
The manuscript submitted is a protocol for a systematic review, therefore not applicable.
X27-i) State the relation of the study team towards the system being evaluated
All authors have reported no potential conflicts of interest existing with any organisations concerning issues discussed in this article.
